# Narrative reversals and story success

**DOI:** 10.1126/sciadv.adl2013

**Published:** 2024-08-21

**Authors:** Samsun Knight, Matthew D. Rocklage, Yakov Bart

**Affiliations:** ^1^ University of Toronto, Toronto, ON, Canada.; ^2^ DATA Initiative at Northeastern University, 360 Huntington Ave, Boston, MA 02115, USA.; ^3^ Northeastern University, Boston, MA 02115, USA.

## Abstract

Storytelling is a powerful tool that connects us and shapes our understanding of the world. Theories of effective storytelling boast an intellectual history dating back millennia, highlighting the significance of narratives across civilizations. Yet, despite all this theorizing, empirically predicting what makes a story successful has remained elusive. We propose narrative reversals, key turning points in a story, as pivotal facets that predict story success. Drawing on narrative theory, we conceptualize reversals as plot: essential moments that push narratives forward and shape audience reception. Across 30,000 movies, TV shows, novels, and fundraising pitches, we use computational linguistics and trend detection analysis to develop a quantitative method for measuring narrative reversals via shifts in valence. We find that stories with more‚ and more dramatic, turning points are more successful. Our findings shed light on this age-old art form and provide a practical approach to understanding and predicting the impact of storytelling.

## Teaser

Combining computational linguistics and classical dramaturgy, we introduce a way to measure plot and predict narrative impact.

## INTRODUCTION

Storytelling is universal. It exists in every known culture and its origins stretch back further than what writing can capture (*1*, *2*). Through stories, we connect, share values, and entertain each other. We tell tales to explain our past and to imagine our future (*3*, *4*). We spin stories to persuade others and evoke emotion. Whether in movies, TV shows, novels, or everyday conversations, the stories we tell each other can enchant and enthrall us.

But while some stories captivate us and achieve lasting acclaim, others bore us and are universally panned. How can we predict which stories will disappoint and which will go on to be sensations?

The puzzle of what makes for a compelling narrative has engaged scholars for millennia. Throughout this time, numerous philosophers and dramaturgs have offered invaluable insights into the world of storytelling. However, testing these insights has been challenging, as it requires identifying diverse narratives, systematically quantifying them, and then tracking their subsequent success.

### Computational linguistics and dramaturgical theory

Recent advances in computational linguistics offer an unprecedented opportunity to evaluate and build on accumulated wisdom (*5*). Yet, recent computational work grounded in dramaturgical theory has largely been unable to identify predictors of the impact of narratives. For example, dramaturgs have long hypothesized there is a core set of narrative patterns (e.g., rise-fall, rise-fall-rise, etc.) and that these patterns shape the impact of the story (*6*–*8*). While researchers were able to identify these basic patterns using automated clustering analysis, they did not predict success (*9*). Similarly, the influential 19th-century writer and scholar Gustav Freytag proposed three elements pivotal to narratives: staging, plot progression, and cognitive tension (*6*). However, adherence to or deviation from these elements also has not predicted a narrative’s success (*10*). Likewise, researchers built measures to detect the “most reportable events” (i.e., the most salient event) in a given piece of narrative text by quantifying changes in linguistic complexity, meaning, and emotion, based principally on the ideas of literary theorist Gerald Prince (*11*), but again presented no relationship to story success (*12*). Thus, it is still unclear how humanity’s accumulated knowledge of narratives can help understand what makes for a compelling narrative.

Adding to this dilemma, recent research that has successfully predicted a narrative’s impact has taken a largely atheoretical approach. For example, research has quantified movie scripts to measure how quickly they move (*13*, *14*) and showed that the quicker a script moves, the more favorably it is rated by viewers. While leveraging tools offered by computational linguistics, this research is not based on prior narrative theory.

Overall, this pattern of results raises questions about the validity of accumulated wisdom in predicting audience response. Moreover, the atheoretical nature of metrics that do predict success makes it difficult for scholars to incorporate these findings into a broader conceptual framework. This hinders our ability to advance the understanding of stories as a whole. Can we leverage computational approaches to predict success while also building on past wisdom?

### A different approach: Narrative reversals

In this work, we propose an approach that returns to traditional theories of narratives, but at a more granular level than has previously been assayed. Earlier approaches that relied on dramaturgy did so to classify stories into high-level categories, rather than to measure the constituent elements of narrative that are theorized to drive audience appeal. For instance, as noted previously, research has used automatic clustering to classify narratives as “rags to riches” or “Cinderella” stories and indicated that the vast majority of narratives had plot arcs that fit into exactly six such patterns (*9*). In essence, researchers built a “narrative periodic table” without delving into the fundamental atoms that make it up, skipping forward to classifying broad patterns as an explanation of story success. In doing so, prior work overlooked key theoretical explanations of the fundamental mechanisms of a story’s attention-grabbing appeal. Consequently, it has not shown why some stories that appear to have similar structures fail to evoke positive reactions, while others garner acclaim. While this earlier work made important strides in building this narrative periodic table, the present work uses dramaturgical theory to build more granular measures of narratives to quantify the potential energy, so to speak, of each story.

Specifically, we propose that focusing on narrative reversals as pivotal elements of compelling stories is key to understanding a story’s potential. Narrative reversals, or turning points in a narrative, have long been theorized as integral to moving narratives forward and creating engaging plots. For example, in narratological theories of story structure, reversals, and related ideas like “breaches” from the usual state of affairs are key features that distinguish stories from other text (*11*, *15*–*18*). These theories suggest that narratives with more of these turning points feel more “storylike” and engaging, while those without them feel “pointless” (*15*).

These turning points can be relatively large or small, but, in dramaturgical theory, they crucially represent events that shift the course of the narrative from positive to negative, or vice versa. For example: after Romeo and Juliet fall head-over-heels for each other at the masked ball, only to realize that their love is forbidden by their families; when Llewyn Davis in *Inside Llewyn Davis* wakes up in his friend’s apartment and writes them a nice thank-you note, but then accidentally locks both himself and their cat outside; when the villain in *Iron Man 2* is about to blow the explosive drones and kill Pepper, but Iron Man flies back and saves her just in the nick of time.

The earliest mention of narrative reversals dates to Aristotle, who named peripeteia, or the sudden reversal of circumstances, as one of the defining aspects of the perfect story (*19*). More recently, famed novelist E. M. Forster (*20*) defined a narrative plot as a sequence of events that induce change; this concept was then further refined in the work of Yale dramaturg Leon Katz and screenwriting guru Robert McKee, who specified that such change-inducing events are marked by reversals in a story’s state of affairs from positive to negative, or vice versa (*21*, *22*). Their definition of narrative plot is, precisely, the stringing together of such turning points from positive to negative or negative to positive, which combine into a continuous sequence of events that each cause a manifest change or reversal in the story’s state of affairs (*6*, *20*–*22*). Similarly, novelist Kurt Vonnegut asserted that story movement is best understood by graphing the oscillation between positivity and negativity over the course of the narrative (*23*). Following this theory, we conceptualize narrative reversals as precisely such oscillations.

As an example, returning to Romeo and Juliet, the play’s love-at-first-sight Scene 5 of Act 1 famously turns when Romeo’s new love collides with the Montague-Capulet family feud. This reversal directly sets off the long chain of events that is precisely the plot of the story, from secretly marrying Juliet (positive) to being banished shortly thereafter (negative). By quantifying and tracing such reversals in valence across a narrative, we can identify critical moments and measure their attributes, deepening our understanding of how stories succeed, and offering valuable insights into the universal appeal of compelling narratives.

### Hypotheses

Dramaturgical theory further gives clear prescriptions for the ideal quantity and shape of narrative reversals within stories. First, stories with more reversals that shift from positive to negative or vice versa are theorized to be stories with more plot (more “story events” that “produc[e] emotion in characters and audience alike”) and are therefore more engaging, especially in contrast to stories with flat “event”-less sections without reversal (*22*). Similarly, these reversals can also surprise the audience (*24*), as they can deviate from the expected story path; surprise is thought to be a key factor in audience engagement [see also (*5*, *25*)]. Thus, a greater number of reversals is predicted to lead to a more positive audience response.

Second, dramaturgical theory suggests that reversals are “ideally structured in terms of total opposites, from one state of affairs to its total opposite,” and as such, stories that contain larger, more dramatic reversals are theorized to be more compelling (*21*). Succinctly, then, dramaturgy proposes that there should be “no scene that doesn’t turn…from the positive to the negative or the negative to the positive”: every moment in a good story should either be introducing a new “turn,” increasing reversal quantity, or building to a greater “opposite,” increasing reversal magnitude. Similarly, larger reversals have the potential to create more surprise. On this basis, we propose that the more engaging narrative will have both more and larger turning points, which will manifest as more and larger reversals in the state of affairs.

To test these hypotheses and measure narrative reversals on a large scale, we use computational linguistics to quantify the degree of positivity of narratives as they unfold. This allows us to detect reversals in the story’s affective trajectory, which provides a measure of reversals in the story’s state of affairs. We then apply trend detection analysis to identify narrative reversals and use the number and magnitude of these turning points to predict narrative success. We test the predictive power of these reversals in nearly 30,000 narratives across four domains: movies, TV shows, novels, and GoFundMe fundraising pitches. We evaluate whether stories with more and larger reversals are more highly rated or more popular and whether online fundraising pitches are more likely to succeed.

## STUDY 1: MOVIES

Study 1 uses programmatic text analysis to detect reversals in thousands of movies. We predict that movies with more and larger reversals will be more successful.

### Method

We first collected all movie text from OpenSubtitles.com. These data contain both the movie dialog and action as well as timestamps for when these occur on screen. Most movies were released between 1990 and 2022 and include a wide variety of smaller movies (e.g., *Rumba Love*) and blockbusters (e.g., *Iron Man 2*). These movies cover a broad range of genres from action to comedy to horror. To compare similar movies and ensure a minimum word count for analysis, we constrain our sample to movies with at least 1000 words and that contain a minimum of one word in each 5-min window. Results are similar when inspecting the unrestricted sample (see table S1). There were 3713 movies meeting these criteria for final analyses.

The identification of narrative reversals followed from narrative theory. Specifically, the identifying facet of a narrative reversal is an observable shift in the valence of story (*6*, *19*–*22*). To that end, the affective trajectory of the script was measured as the narrative unfolded. The positivity of the text was computed using the VADER lexicon (*26*), which quantifies words on a scale from extremely negative (−4) to extremely positive (+4). For example, words like “doomed,” “terrifying,” and “disaster” are scored negatively (−3.20, −2.70, and −3.10, respectively), while “lovely,” “charming,” and “happy” have positive scores (2.80, 2.80, and 2.70, respectively). VADER has been shown to correlate well with human ratings and to generalize across contexts (*26*). Results are similar when using other approaches to quantify valence, including the LabMT lexicon (*27*) and the Evaluative Lexicon [(*28*, *29*); see the Supplementary Materials for details]. That said, given their shorter dictionaries, for example, the Evaluative Lexicon focuses on measuring the valence of opinion words, there is nonsignificance in some estimates of the effect of reversal magnitude with these other measures (see the Supplementary Materials for details). This approach allows us to build an empirical analogue to the changing state of affairs in the narrative, as described in the theory.

**Fig. 1.**
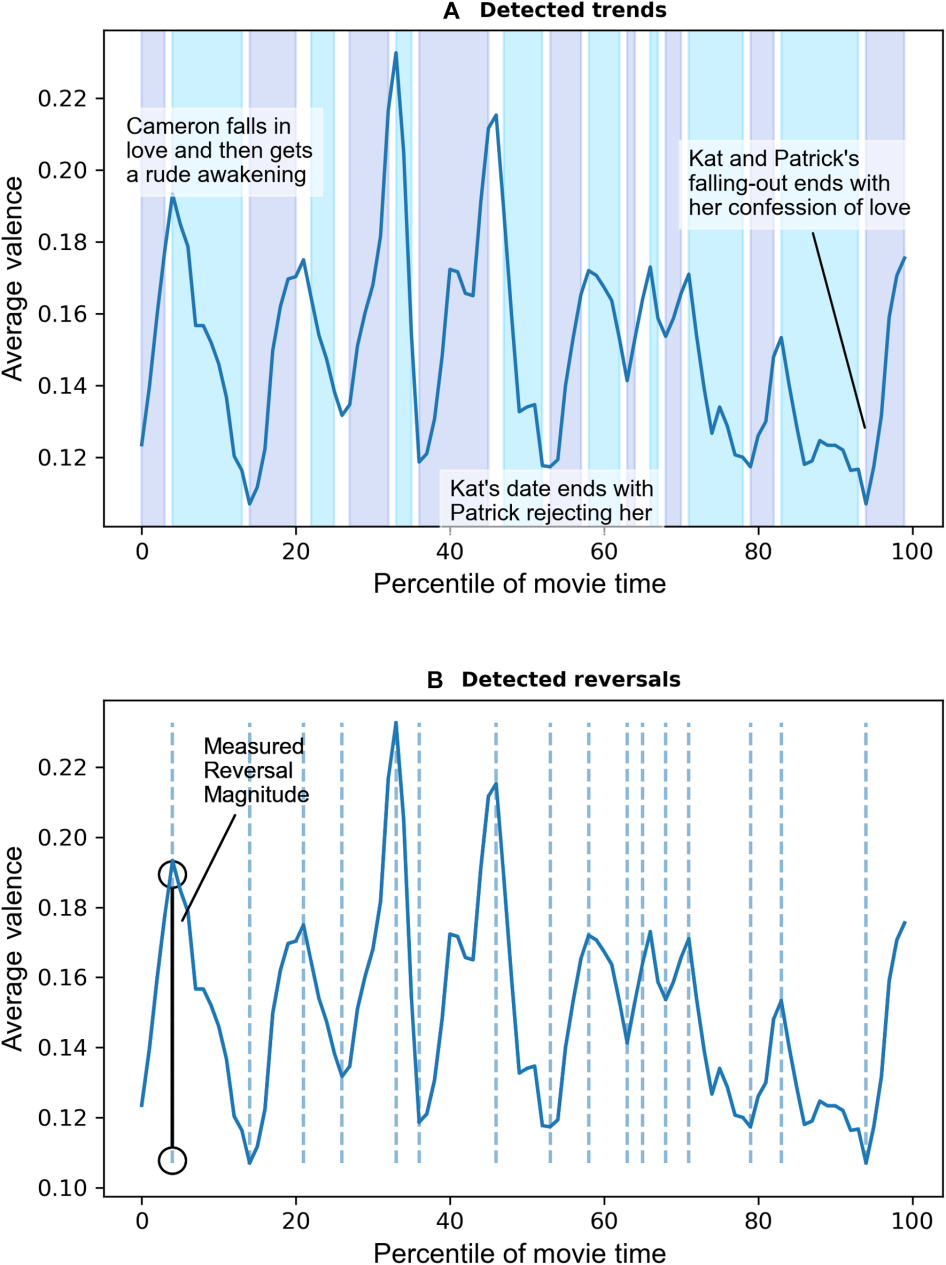
Example illustration of reversal detection for *10 Things I Hate About You*. (**A**) Upward (purple) and downward (blue) trends from *10 Things I Hate About You* movie. (**B**) Identified reversals from *10 Things I Hate About You* movie.

To give a concrete example, in the 1999 romantic comedy, *10 Thing I Hate About You*, the story starts on a positive note, building up to the first reversal. Words in the script like “beautiful” and “love” signify that one of the main characters quickly falls in love with the younger Stratford sister. Yet, the narrative quickly reverses when the older Stratford sister is introduced as a roadblock to this romance. More negative words like “bitch” and “jerk” are used, signifying an obstacle: The younger sister is only allowed to date if her older sister starts dating too (see Fig. 1).

To trace affective trajectories across time, following prior convention (*9*, *30*), we computed the positivity of each of 100 fixed-width windows. Window overlap was standardized so as to maintain the same width and same number of windows across all screen times, following prior convention (*9*, *30*). Results are also similar using alternative window overlaps for this and subsequent studies (see table S2). These moving-average windows were based on subtitle timestamps over a given number of minutes of screen time. This creates averages of valence based on which words occur proximally in the actual movie as it is experienced. Thus, this has the advantage of approximating the pacing of the story as experienced by the audience. We use a 5-min window, but results are similar when inspecting smaller or larger windows (see table S3).

After quantifying the text and creating a raw time series of valence, we identified reversals using the trendet algorithm (*31*). This algorithm identifies upward (downward) trends by detecting segments where each successive point is greater (less) than the trend’s moving average to that point (see the Supplementary Materials for more). This measure of narrative reversals was further validated in an independent experiment (see the Supplementary Materials).

Figure 1 illustrates an example of trend reversals in a movie (*M* = 14.46 and SD = 2.78) and the average reversal magnitude as the average absolute peak-to-trough distance in valence between two successive reversal points (*M* = 0.06 and SD = 0.02). See the Supplementary Materials for additional summary statistics, correlation matrices, and histograms for this and all subsequent studies. Last, following prior research (*13*, *14*, *30*), we measured movie success using the aggregated star ratings from IMDb.com (*M* = 6.44 and SD = 1.02).

### Results

As predicted, movies with more reversals and larger reversals receive higher ratings. Regression results are presented in Table 1. For this and all other results, continuous predictors are standardized and outcome variables are unstandardized. SEs for each model parameter are presented in parentheses in each table.

**Table 1.** Reversals and movie evaluations.

Predictors	Reversals (1)	Controls (2)
Number of reversals	0.166***	0.085***
	(0.019)	(0.019)
Average reversal magnitude	0.039*	0.101***
	(0.019)	(0.023)
**Controls**		
Average valence		−0.186***
		(0.020)
Budget		0.145***
		(0.021)
Subtitle word count		0.302***
		(0.020)
Semantic circuitousness		−0.099***
		(0.022)
Semantic volume		−0.194
		(0.115)
Semantic speed		0.327**
		(0.122)
Sentiment volatility		0.059**
		(0.021)
Year fixed effects	NO	YES
Genre fixed effects	NO	YES
Maturity rating fixed effects	NO	YES
Constant	6.447***	8.249***
	(0.017)	(0.855)
Adjusted *R*^2^	0.021	0.314
No. of movies	3713	3713

*P < 0.05**P < 0.01,***P < 0.001.

To better identify the unique effect of reversals, we tested whether the results are robust to the inclusion of a large battery of controls. First, we include measures of narrative features from prior research to control for other measures that have been shown to predict narrative success: sentiment volatility, semantic speed, semantic circularity, and semantic volume (*13*, *14*, *30*). It is possible that narrative reversals pick up on one of these metrics, and thus we control for them to assess this possibility.

Second, we control for word count to ensure that our results are not driven by movie length. Perhaps longer movies have more opportunity for complex narratives and movie length better accounts for the predictive effect of reversals.

Third, we control for budget. Higher-budget movies often have better production quality, marketing, and star power, and these factors could account for the effect of reversals.

Fourth, we control for the average valence of the script to control for the general affective tone of the movie. It is possible that the tone of the movie better accounts for the effect of reversals.

Fifth, we also include fixed effects of movie year. Movie preferences and expectations change over time due to cultural trends and technological advancements, and perhaps release year accounts for the effect of reversals.

Sixth, we include fixed effects for movie genre. Different movie genres have distinct conventions, audience expectations, and success criteria, and genre could account for reversal’s effect.

Seventh, we include fixed effects of movie maturity rating (e.g., PG and PG-13). Movies with different maturity ratings have different target audiences and content restrictions, and the maturity rating might account for the effect of reversals. However, even after including all of these controls, the number and magnitude of reversals significantly predict more positive movie ratings (see Table 1).

We also considered “peak/end” effects, the idea that experiences are judged by their most intense (peak) and final (end) moments (*32*), to examine whether those could better account for the effects of reversals. However, there were no significant effects of the peak [*B* = −0.017, *t*(3708) = −0.80, and *P* = 0.43] or end reversal magnitudes [*B* = −0.026, *t*(3708) = −1.38, and *P* = 0.17]. Moreover, the number of reversals and the average magnitude of reversals remained significant (*P*_num_ < 0.001 and *P*_mag_ = 0.01). These results are similar across all subsequent studies and thus peak/end effects cannot account for the observed effects (see the Supplementary Materials for results across studies).

We also examined the boundary on the effect of the number of reversals. Is it possible to have too many reversals in a narrative? To test this, we added a squared term to the model for the number of reversals. However, the result suggests that the effect of the number of reversals accelerates as it grows [*B* = 0.427, *t*(3784) = 3.925, and *P* < 0.001]. Moreover, this finding does not replicate in the subsequent studies and there is no consistent effect across studies (see the Supplementary Materials for results across studies). We also used a bottom-up, nonparametric approach with generalized additive modeling to flexibly model the data without imposing a specific functional form. This approach showed a similar pattern (see fig. S3). Given this, the current evidence does not suggest a curvilinear effect observable in the data, although it is possible this asymptotic effect would appear in some contexts. Nevertheless, an important caveat is that we only observe data for movies that were brought to market, so it is plausible that there is an upper limit to the number of reversals in narrative. Still, there was no consistent discernable asymptotic effect for the success of 30,000 movies, TV shows, novels, or fundraising pitches, suggesting that if such an asymptote exists, it was not observable even with a large sample of stories.

To make the effect of reversals more concrete, going from the fewest number of observed reversals to the most would result in a roughly 1.4 star increase in IMDb rating. For context, on the basis of our point estimates, this corresponds to the same expected increase in star rating as a roughly $40 million increase in movie budget. Similarly, going from the smallest average observed reversal magnitude to the largest would result in a roughly 0.4 star increase in IMDb rating. On the basis of our point estimates, this would be comparable to the effect of a roughly $12 million increase in budget.

## STUDY 2: TV SHOWS

Study 1 found that movies with more and larger reversals were more successful. Study 2 uses programmatic text analysis to detect reversals in tens of thousands of TV episodes. This study allows us to test the generalizability of the effects across domains, for media of different length of content, and narratives of a more episodic nature. Given that TV episodes also often come from the same series (e.g., *The Simpsons*), study 2 also provides the opportunity to hold additional features relatively constant, such as the tone and style of the show, its general theme, the quality of the writers, and the demographics of typical audience members. Beyond these features, we predict that episodes with more reversals and shows with larger reversals are more successful.

### Method

We used the same approach as in study 1. Subtitles data come from the same source, OpenSubtitles.com, and with the same information. Most TV episodes were aired between 1990 and 2022 and span from relatively obscure TV shows (*The Venture Bros*.) to very well-known series (*The Simpsons*). We again constrain our analysis to scripts with at least 1000 words and at least one word in each moving window. Results are similar when inspecting the unrestricted sample (see table S6). The final sample consists of 19,339 TV episodes, with genres from talk shows to thrillers to science fiction.

As TV episodes are generally shorter than movies, we use a 3-min moving window of duration to compute percentile valence averages, but results are similar when using the same 5-min window as above or a smaller window (see table S7). Then, we compute the number of reversals (*M* = 11.91 and SD = 2.91) and average magnitude of reversals (*M* = 0.06 and SD = 0.02) for each episode using the same trendet algorithm and match these to TV episode IMDb ratings (*M* = 7.77 and SD = 0.72).

### Results

As predicted, TV episodes with a greater number of reversals and with larger reversals receive higher ratings. Regression results are presented in Table 2.

**Table 2.** Reversals and TV evaluations.

Predictors	Reversals (1)	Controls (2)	Controls (3)
Number of reversals	0.039***	0.035***	0.019**
	(0.006)	(0.008)	(0.006)
Average reversal magnitude	0.030***	0.038***	0.024***
	(0.006)	(0.009)	(0.007)
**Controls**			
Average valence		−0.069***	−0.069***
		(0.007)	(0.006)
Subtitle word count		−0.020*	−0.014
		(0.008)	(0.008)
Semantic circuitousness		−0.083***	−0.032***
		(0.010)	(0.008)
Semantic volume		−0.348***	−0.304***
		(0.050)	(0.044)
Semantic speed		0.427***	0.320***
		(0.054)	(0.048)
Sentiment volatility		0.002	0.025***
		(0.008)	(0.007)
TV show fixed effects	NO	NO	YES
Year fixed effects	NO	YES	YES
Genre fixed effects	NO	YES	YES
Maturity rating fixed effects	NO	YES	YES
Constant	7.767***	8.691***	7.843***
	(0.005)	(0.675)	(0.415)
Adjusted *R*^2^	0.003	0.132	0.547
No. of TV episodes	19339	19339	18686

*P < 0.05**P < 0.01***P < 0.001.

As in study 1, to test alternative explanations, first, we include the same measures of narrative features from prior research to control for other measures that have been shown to predict narrative success (e.g., sentiment volatility). Second, we include controls for word count to ensure that our results are not driven by episode length. Third, we control for the average valence of the script to control for the general affective tone of the episode. Fourth, fifth, and sixth, we include fixed effects of release year, genre, and maturity rating (e.g., PG and PG-13). However, even after including all of these controls, the number and magnitude of reversals significantly predict more positive TV episode ratings (see Table 2).

Last, we also test a specification that includes fixed effects for individual TV series. This allows us to compare episodes within the same series (e.g., *Yellowstone*, *Xena Warrior Princess*, and *The Simpsons*), which addresses an important concern: It could be that some series have specific qualities that lead them to be more successful, e.g., style, theme, writers, and audiences, and also just tend to contain more or larger reversals. Even controlling for series, we continue to find a significant relationship between reversal measures and episode success (see Table 2, model 3). For example, *Yellowstone* episodes with more and larger reversals are more successful than other episodes of the same show. This indicates the reversal-success link is not solely driven by intrinsic differences between shows but also holds for episodes within the same series.

To make the effect of reversals more concrete, going from the fewest number of observed reversals to the most would result in a roughly 0.35 star increase in IMDb rating. Similarly, going from the smallest average observed reversal magnitude to the largest would result in a roughly 0.30 star increase in IMDb rating.

## STUDY 3: NOVELS

Study 3 uses programmatic text analysis to measure reversals in thousands of novels. This allows us to further evaluate the generalizability of the effects of reversals against a third narrative domain and moreover, one that consists only of the printed word. While we have provided evidence that reversals predict positive evaluations in screen media, both movies and TV shows contain visuals and sounds, which we cannot entirely account for in our data. Investigating a text-only medium allows us to establish that unobserved audiovisual components are not driving factors in our observed effect. In this context, however, IMDb ratings are unavailable, so we use alternative measures of story success to evaluate the relationship between reversals and story outcomes: popularity. As before, we predict that novels with more reversals and larger reversals will be more successful.

### Method

We collected all texts from Project Gutenberg, using the standardized dataset of Gerlach and Font-Clos (*33*). These data contain thousands of full manuscript texts, such as *Middlemarch*, *Little Women*, and *Dracula*, with most of the sample coming from authors born between 1800 and 1920. Given our focus on narratives, we restricted our attention to English-language novels, defined as works categorized under “fiction” or “literature” and that contain at least 50,000 words, the standard industry definition of novel length (*34*–*36*). Results are also similar when using a smaller word-count threshold (see table S11). Following these criteria, there was a final sample of 8663 English-language novels, with genres spanning from war novels to romance novels to fantasy novels.

We perform the same time-series analysis as the previous studies and construct measures of the number of reversals in each novel (*M* = 9.92 and SD = 3.14) and the average magnitude of reversals (*M* = 0.02 and SD = 0.01). Following convention for novels (*9*), we used 10,000-word-count moving windows across the length of the manuscript. Results are similar using a smaller or larger word-count window (see table S12) and keeping overlap between windows fixed across novel lengths (see table S13).

To measure the popularity of a novel, we used the log number of downloads from the Gutenberg website (*M* = 3.09 and SD = 1.19), as reported in the Standardized Project Gutenberg corpus data (*33*). We present this as a measure of the enduring popularity of the works in the present day.

### Results

As predicted, books with more reversals and with larger reversals are more popular. Regression results are presented in Table 3.

**Table 3.** Reversals and novel downloads.

Predictors	Reversals (1)	Controls (2)	Controls (3)
Number of reversals	0.166***	0.074***	0.067***
	(0.015)	(0.019)	(0.019)
Average reversal magnitude	0.156***	0.086***	0.079***
	(0.015)	(0.020)	(0.020)
**Controls**			
Average valence		−0.185***	−0.187***
		(0.014)	(0.014)
Novel word count		0.071***	0.068***
		(0.019)	(0.019)
Semantic circuitousness		0.026	0.018
		(0.028)	(0.028)
Semantic volume		0.320**	0.273*
		(0.107)	(0.106)
Semantic speed		−0.349***	−0.294**
		(0.095)	(0.094)
Sentiment volatility		0.145***	0.138***
		(0.024)	(0.024)
Author decade-of-birth fixed effects	NO	NO	YES
Genre fixed effects	NO	YES	YES
Constant	3.084***	3.205***	3.172***
	(0.013)	(0.055)	(0.054)
Adjusted *R*^2^	0.018	0.130	0.158
No. of novels	8663	8663	8663

*P < 0.05**P < 0.01***P < 0.001.

As in the prior studies, to test alternative explanations, first, we include measures of narrative features from prior research to control for other measures that have been shown to predict narrative success (e.g., sentiment volatility). Second, we include controls for word count to ensure that our results are not driven by novel length. Third, we control for the average valence of the script to control for the general affective tone of the novel. Fourth and fifth, we include fixed effects of approximate publication year (author’s decade of birth) and genre. We use author decade of birth as a stand-in for publication date as the Gutenberg dataset does not provide a publication date for each work. Last, one may worry that unrelated stylistic differences over different eras or story types may correlate with both reversal measures and popularity, so we control for both with these measures. However, even after including all of these controls, the number and magnitude of reversals significantly predict popularity (see Table 3).

To make the effect of reversals more concrete, going from the fewest number of observed reversals to the most would result in a roughly 110% increase in the number of downloads. Similarly, going from the smallest average observed reversal magnitude to the largest would result in a roughly 160% increase in the number of downloads.

## STUDY 4: FUNDRAISING PITCHES

Results of studies 1, 2, and 3 are consistent with our theorizing, but one may be concerned that the above are all contexts where professional writers are presenting honed narratives in entertainment contexts and therefore, that the results may not generalize more widely. Yet, people tell each other stories on a daily basis. Extending the results to amateur stories can help inform the power of narrative reversals across people and contexts.

To that end, study 4 uses programmatic text analysis to detect reversals in more than a thousand GoFundMe fundraising pitches. GoFundMe.com is a prominent fundraising platform that allows users to post descriptions of their financial needs and to petition friends, family, and strangers online to help raise money to reach their stated fundraising goal. For example, one person sought financial support for her friend who was in a debilitating car accident, leaving him unable to work. Another person sought aid to support her pilgrimage to a religious festival.

This context is ideal for exploring a setting where amateurs attempt to tell their own story: from GoFundMe’s own writing tips, posters are instructed that “compelling stories turn passive visitors into active donors and sharers,” and are encouraged to write their fundraising pitches as engaging narratives (*37*). Thus, according to the website itself, the extent to which posters are able to achieve this is predicted to be instrumental to their potential success. We predict that GoFundMe pitches with more reversals and larger reversals will have a higher probability of reaching their fundraising goal.

### Method

We collected data on thousands of GoFundMe fundraisers. As GoFundMe’s search function returns only a subset of possible fundraisers, we used a simple approach to trawl for as many fundraisers as possible in a transparent manner: using the Social Security list of the top 100 most popular men’s names and top 100 most popular women’s names in the US in the GoFundMe search bar (*38*). We gathered data for all fundraisers, both completed and in progress, that were returned through each of these 200 keyword-name searches.

As with our prior studies, we restrict consideration to fundraisers with pitches of 1000 words or more to ensure sufficient text to build the moving-window measures. In addition, we required that GoFundMe campaigns must have been posted for more than 2 weeks to allow for a minimum of time passed to reach fundraising goals. Results are similar when considering campaigns that have been posted for 30 days or more (see table S16). Following these criteria, the final sample consists of 1133 fundraising pitches, from medical fundraisers to education fundraisers to fundraisers for funerals and memorials. Each pitch’s webpage also contained information on the poster’s fundraising goals and fundraising totals. Whether a pitch met its monetary goal (coded as “1”) or not (“0”) provided the measure of success.

Following convention for shorter texts (*30*), 500-word-count moving windows were used to construct the pitches’ time series of valence. Using the same trendet algorithm, we compute measures of the number (*M* = 7.38 and SD = 3.10) and magnitude of reversals (M = 0.03 and SD *=* 0.02) for each fundraiser. Results are similar using smaller or larger window sizes or keeping window overlaps fixed across different fundraiser lengths, although not as consistently significant (see tables S19 and S20).

### Results

As predicted, fundraising pitches with more reversals and with larger reversals are more likely to reach their fundraising goals. Logistic regression results are presented in Table 4.

**Table 4.** Reversals and fundraiser success.

Predictors	Reversals (1)	Controls (2)
Number of reversals	0.271*	0.367*
	(0.116)	(0.151)
Average reversal magnitude	0.320**	0.375*
	(0.107)	(0.154)
**Controls**		
Average valence		0.128
		(0.145)
Fundraiser word count		−0.013
		(0.217)
Number of months online		0.226**
		(0.081)
English language		−0.278
		(0.335)
Semantic circuitousness		−0.093
		(0.275)
Semantic volume		0.215
		(0.595)
Semantic speed		−0.086
		(0.591)
Sentiment volatility		−0.079
		(0.192)
Category fixed effects	NO	YES
Constant	−2.125***	−0.751
	(0.097)	(1.205)
No. of fundraisers	1133	1133
Pseudo *R*^2^	0.011	0.067

*P < 0.05**P < 0.01***P < 0.001.

As in the prior studies, to test alternative explanations, first, we include the same measures of narrative features from prior research to control for other measures that have been shown to predict narrative success (e.g., sentiment volatility). Second, we control for word count to ensure that our results are not driven by pitch length. Third, we control for the log of the fundraising goal to show that our results are not driven by particular fundraising goals. Fourth, we control for the time duration that the fundraiser has been online to show that results are not driven by certain pitches staying online for longer. Fifth, we control for the average valence of the fundraiser to control for the general affective tone of the fundraising pitch. Sixth and seventh, we include fixed effects to control for fundraising category and the language used (e.g., English). However, even after including all of these controls, the number and magnitude of reversals significantly predict more positive fundraising pitches (see Table 4).

To make the effect of reversals more concrete, going from the fewest number of observed reversals to the most would result in a roughly 39 percentage point increase in the probability of success. Similarly, going from the smallest average observed reversal magnitude to the largest would result in a roughly 49 percentage point increase in the probability of success.

## DISCUSSION

Quantitative research into the nature of narratives and the determinants of their success has made leaps and strides in recent years. Recent applications of computational linguistics have enabled researchers to build quantitative measures of narrative, and vast new data sources have allowed for the construction and testing of such measures at scale. However, to date, research has been bifurcated. Approaches that leverage narrative theory have been unable to gain traction in predicting success, leaving narrative theory without an engine of predictive power to drive it forward. Meanwhile, approaches focusing on predicting success lack a theoretical basis, leaving them rudderless.

In this work, we present a computational method to leverage theory to develop measures of fundamental narrative mechanisms, in this case, trend reversals that measure story turning points, the building blocks of plot, to rejoin these literatures and provide a path forward for future research. By leveraging theory to provide insights into the more granular workings of stories, we develop a way to build theory-guided measures that are both grounded in dramaturgy and predict story success across a wide set of domains, from movies to TV to novels to online amateur fundraising pitches. We detect narrative reversals using reversals in the affective trajectory of the texts using three different linguistic measures and find converging evidence: stories with more and with larger, more dramatic reversals, were more successful, even when subject to a wide battery of controls to provide evidence against alternative explanations.

The results of this research underscore the importance of dramaturgical theory and further enrich it with empirical evidence. By quantifying the concept of reversals, we provide support for dramaturgical theory, which has long highlighted the critical role of turning points in shaping narrative structure and appeal. Indeed, the quantification of reversals and their link to narrative success serves to validate and augment long-standing theoretical tenets, enhancing their applied relevance. Rather than viewing dramaturgical theory as abstract or detached from real-world outcomes, we show that its concepts can be used to predict the success of a narrative in a measurable way.

This work points the way forward to an emerging line of research that seeks to use theory to build more granular measures of narrative flow and mechanisms, and shows that developing measures of such component mechanisms provides a fruitful path to successfully joining narrative theory with empirical work. Here, we develop measures of narrative reversals, one of the key ingredients of a compelling plot, and demonstrate that measurements of the attributes of a story’s reversals allow us to measure key components of the story’s appeal. Future work may consider more detailed reversal attributes apart from the quantity and magnitude attributes that we consider here; for example, future research could consider the regularity of reversal pacing over the course of a narrative, the variability in reversal magnitudes, or whether reversals increase or decrease in magnitude over the course of a tale. By detecting reversals as a measurable ingredient of narrative, this work opens a whole domain of reversal-based analyses of narrative forms.

The current research also has broader implications for society as a whole. Narratives hold a unique place in our social fabric, serving as tools to shape our understanding of the world, convey cultural and moral values, and influence our behaviors. By understanding what makes stories compelling, we gain insight into the human condition and the cognitive structures that guide our reactions and emotions. This work, therefore, is not just about defining what makes a good story; it extends to informing how narratives can educate, inform, and inspire individuals and communities.

Moreover, these findings have wide-ranging implications for practitioners in various fields where narratives play a pivotal role. For filmmakers, authors, and novices alike, the quantified insight into the importance of reversals offers a robust tool to enhance the structure of their stories. Our findings can help guide creators to craft more compelling narratives by introducing frequent and dramatic turning points, help creative industry professionals evaluate and develop new story drafts, and help charities and individuals to write more effective fundraising appeals. Understanding the power of narrative reversals and other key narrative ingredients allows us to harness the persuasive power of storytelling more effectively, be it in education, politics, marketing, or other fields where narratives play a crucial role. Consequently, this research has the potential to provide not just theoretical advancements but also practical tools that have broader implications across domains.

All the same, while this work pushes forward our understanding of narrative and introduces a powerful practical tool, there remain certain limitations that are outside of the scope of this paper to address, but that we hope future work may tackle. The current studies share the limitations of all field studies in that they cannot definitively conclude causality. While the observed effects persist when assessing a number of possible alternative explanations, there may remain other omitted variables or model misspecification. We also present evidence that the measures capture narrative turning points as experienced by readers, and leverage a long lineage of narrative theorizing to interpret our measures as capturing plot as such. While these measures are necessarily approximate, for example, good things may happen to bad people within a narrative, which may be difficult to fully capture, we expect such noise in measurement would likely attenuate our results, suggesting that our findings here could be a conservative estimate of the true effect of reversals. Still, future research can work to further identify the unique effect of reversals via controlled experiments where different facets of a narrative are held constant while altering the number and magnitude of reversals.

Future work could also explore the mechanisms behind narrative reversals. As noted earlier, dramaturgical theory has strong predictions for the importance of such reversals, in particular linking the larger quantity and magnitude of such reversals to the experience of “plot tension” (*21*), the “hold upon [the audience’s] nerves” (*6*), the periodic release of “revelation” (*5*), or the degree of suspense/surprise (*24*, *25*). Investigating the psychological mechanisms that lead to feeling “gripped” by a narrative and how they link to narrative reversals would be a fruitful and promising avenue for subsequent work. This research therefore provides a springboard for future work where researchers can investigate the specific ways that reversals drive audience engagement and favorability of response.

Storytelling is both an art and a science, and one of the most ancient of either that humans have. Many of the greatest thinkers of history have developed powerful theories for why stories affect us so profoundly and how to craft the most engaging narratives for audiences. Here, we develop one set of measures to capture these insights and apply them in a modern scientific application, to enrich our present understanding and develop practical tools for understanding stories. We hope that this introduces a new domain for analyzing and understanding narratives and serves to exemplify a concrete way to draw from this deep wealth of human expertise.

## Supplementary Materials

### This PDF file includes:

Supplementary Text Tables S1 to S21 Figs. S1 to S12 References
