# Supplementary material for: Narrative reversals and story success
Source: Sci Adv. 2024 Aug 21;10(34):eadl2013. doi: 10.1126/sciadv.adl2013 (PMC11421681; doi:10.1126/sciadv.adl2013)
Supplement: Supplementary file 1 — Supplementary Text Tables S1 to S21 Figs. S1 to S12 References [file sciadv.adl2013_sm.pdf]

Science Advances
AAAS

Supplementary Materials for

Narrative reversals and story success

Samsun Knight *et al.*

Corresponding author: Samsun Knight, samsundknight@gmail.com

Sci. Adv. **10**, eadl2013 (2024)
DOI: 10.1126/sciadv.adl2013

This PDF file includes:

Supplementary Text
Tables S1 to S21
Figs. S1 to S12
References

Supplementary Text

Study 1: Movies

Methods

Details on Trendet Reversal Measure Construction

To compute reversals, first, we split the words into 100 overlapping windows, based on a fixed window size, and computed the valence of each window using VADER's ratio score (number of positive words / (number of positive + neutral + negative words)). The degree of overlap of the adjoining windows is dynamically adjusted, depending on the length of the text or the time duration, to fit 100 windows of that same window size. To facilitate the detection of reversals and to avoid false-positive reversals we then further smooth this emotional arc by taking a 3-point moving average of every point in this time series, computing point *i* as the average of points *i*, *i*-1, and *i*+1.

Next, we detect upwards and downwards trends using the trendet algorithm, which defines upwards (downwards) trends as segments where the moving average up-to-that-point exceeds (does not exceed) the subsequent point (*31*). We designate "reversal points" as endpoints of upwards or downwards trends. To ensure that the algorithm detects trend endpoints that occur close to the end, we extend the sentiment time series (only for this step) with a flat section of 20 extra percentiles all at the value of the final percentile. We then remove these extra percentiles after trend detection. In most cases the endpoint of a trend is also right before the start point of the subsequent trend; for segments where this is not the case and there is no labeled trend between two same-direction trends, we also consider the beginning of the second trend (following the unlabeled area) to be a reversal point.

Finally, we compute the number of reversals as the number of detected reversal points, and measure the absolute difference in valence between subsequent reversal points and their immediately adjacent points for all pairs of subsequent reversal points in the time series, including time series endpoints, computing the average absolute difference in valence between subsequent reversal peaks and troughs as the average reversal magnitude.

Results

Alternative Measures of Valence

Results are similar when using alternative approaches to scoring valence, including LabMT (*27*) and the Evaluative Lexicon (*28*, *29*). Each of these alternative approaches, however, relies on relatively shorter dictionaries of words compared to VADER. While this is advantageous in some contexts, such as when focusing on measuring opinions as is the case for the Evaluative Lexicon (*39*), for the current purposes this leaves specific windows with comparatively fewer words to base valence measurements on. This has the effect of leading to smaller sample sizes and greater noise in reversal measurement. Results are nonetheless similar when using LabMT ($B_{num} = 0.221$, $t_{num}(3551) = 12.06, p_{num} < 0.001$; $B_{mag} = 0.017, t_{mag}(3551) = 0.94, p_{mag} = 0.35$) and the Evaluative Lexicon to score valence for movies ($B_{num} = 0.209$, $t_{num}(1997) = 8.26, p_{num} < 0.001$; $B_{mag} = 0.005, t_{mag}(1997) = 0.21$, $p_{mag} = 0.83$), although the effects of reversal magnitude are non-significant.

Extreme Observations

In the histogram below, there is a small number of extreme observations. Results are similar when winsorizing these observations, defined as those three or more standard deviations from the mean ($B_{num} = 0.170$, $t_{num}(3710) = 8.82, p_{num} < 0.001$; $B_{mag} = 0.040, t_{mag}(3710) = 2.00, p_{mag} = 0.046$).

Cross-correlations

We present cross-correlations in Appendix Figure S2. As evidenced in this figure, there is evidence of possible collinearity between semantic speed and volume from Toubia et al. (*13*), something that is also noted in their original paper. Although these variables are not the primary interest of this paper, we nonetheless present specifications in Appendix Table S4 that exclude either semantic speed or semantic volume. All reversal measures remain significant.

Appendix Table S1

Reversals and Movie Evaluations, Unrestricted Sample

Predictors	Reversals (1)	Controls (2)
Number of reversals	0.157***	0.081***
	(0.019)	(0.019)
Average reversal magnitude	0.036^{+}	0.095***
	(0.019)	(0.022)
**Controls**		
Average valence		-0.186***
		(0.020)
Budget		0.146***
		(0.021)
Subtitle wordcount		0.304***
		(0.020)
Semantic circuitousness		-0.097***
		(0.022)
Semantic volume		-0.194
		(0.117)
Semantic speed		0.339**
		(0.125)
Sentiment volatility		0.066**
		(0.020)
Year Fixed Effects	NO	YES
Genre Fixed Effects	NO	YES
Maturity Rating Fixed Effects	NO	YES
Constant	6.448***	8.228***
	(0.017)	(0.855)
Adjusted R-squared	0.019	0.316
N. of movies	3727	3727

NOTES — *** $p < 0.001$, ** $p < 0.01$, * $p < 0.05$, $^{+}$ $p < 0.10$

Appendix Table S2

Reversals and Movie Evaluations, Fixed Window Overlap

Number of reversals	0.082***
	(0.019)
Average reversal magnitude	0.078***
	(0.023)
**Controls**	
Average valence	-0.168***
	(0.020)
Budget	0.153***
	(0.020)
Subtitle wordcount	0.309***
	(0.020)
Semantic circuitousness	-0.105***
	(0.022)
Semantic volume	-0.231*
	(0.114)
Semantic speed	0.373**
	(0.120)
Sentiment volatility	0.044*
	(0.020)
Year Fixed Effects	YES
Genre Fixed Effects	YES
Maturity Rating Fixed Effects	YES
Constant	8.224***
	(0.856)
Adjusted R-squared	0.312
N. of movies	3760

NOTES — *** $p < 0.001$, ** $p < 0.01$, * $p < 0.05$

Window overlap fixed at the median overlap of the baseline specification.

Appendix Table S3

Reversals and Movie Evaluations, Alternative Window Sizes

Window sizes	Smaller (4 min)	Larger (10 min)
Number of reversals	0.078***	0.062**
	(0.018)	(0.021)
Average reversal magnitude	0.083***	0.065**
	(0.022)	(0.025)
**Controls**		
Average valence	-0.183***	-0.185***
	(0.020)	(0.020)
Budget	0.143***	0.145***
	(0.021)	(0.020)
Subtitle wordcount	0.304***	0.297***
	(0.020)	(0.020)
Semantic circuitousness	-0.105***	-0.102***
	(0.022)	(0.022)
Semantic volume	-0.212	-0.191
	(0.115)	(0.113)
Semantic speed	0.350**	0.323**
	(0.122)	(0.120)
Sentiment volatility	0.056**	0.093***
	(0.020)	(0.022)
Year Fixed Effects	YES	YES
Genre Fixed Effects	YES	YES
Maturity Rating Fixed Effects	YES	YES
Constant	8.164***	8.107***
	(0.855)	(0.854)
Adjusted R-squared	0.313	0.314
N. of movies	3639	3800

NOTES — *** p < 0.001, ** p < 0.01, * p < 0.05

Appendix Table S4

Reversals and Movie Evaluations, Dropping Collinear Controls

	No "Speed" Control	No "Volume" Control
Number of reversals	0.083***	0.084***
	(0.019)	(0.019)
Average reversal magnitude	0.103***	0.102***
	(0.023)	(0.023)
**Controls**		
Average valence	-0.188***	-0.186***
	(0.020)	(0.020)
Budget	0.143***	0.144***
	(0.021)	(0.021)
Subtitle wordcount	0.290***	0.297***
	(0.020)	(0.020)
Semantic circuitousness	-0.057***	-0.074***
	(0.015)	(0.016)
Semantic volume	0.109***	.
	(0.019)	(.)
Semantic speed	.	0.123***
	(.)	(0.020)
Sentiment volatility	0.064**	0.061**
	(0.021)	(0.021)
Year Fixed Effects	YES	YES
Genre Fixed Effects	YES	YES
Maturity Rating Fixed Effects	YES	YES
Constant	8.283***	8.261***
	(0.855)	(0.855)
Adjusted R-squared	0.313	0.314
N. of movies	3713	3713

NOTES — *** p < 0.001, ** p < 0.01, * p < 0.05

Appendix Table S5

Summary Statistics for Movie Sample

	Mean	Std. Dev.	Median
Average reversal magnitude	0.0619	0.0182	0.0599
Number of reversals	14.4584	2.7798	14.0000
Budget	30.5701	40.4170	15.0000
Average valence	0.1332	0.0299	0.1325
Wordcount	9.1827	3.4928	8.7160
Semantic circuitousness	0.1277	0.0206	0.1246
Semantic volume	0.2951	0.0584	0.2864
Semantic speed	0.5462	0.1056	0.5301
Sentiment volatility	0.0308	0.0088	0.0293

NOTES — Unstandardized summary statistics for linear covariates in movie sample used in Study 1, based on 3,713 movies. Wordcount measured in units of 1000 words, budget measured in units of millions of dollars.

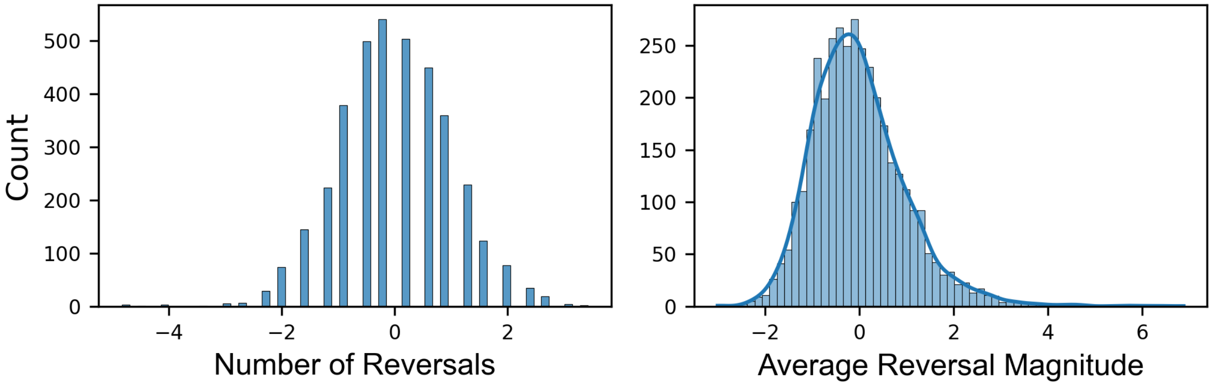

Appendix Figure S1—Histograms of standardized number of reversals and standardized average reversal magnitude for movie sample used in study 1.

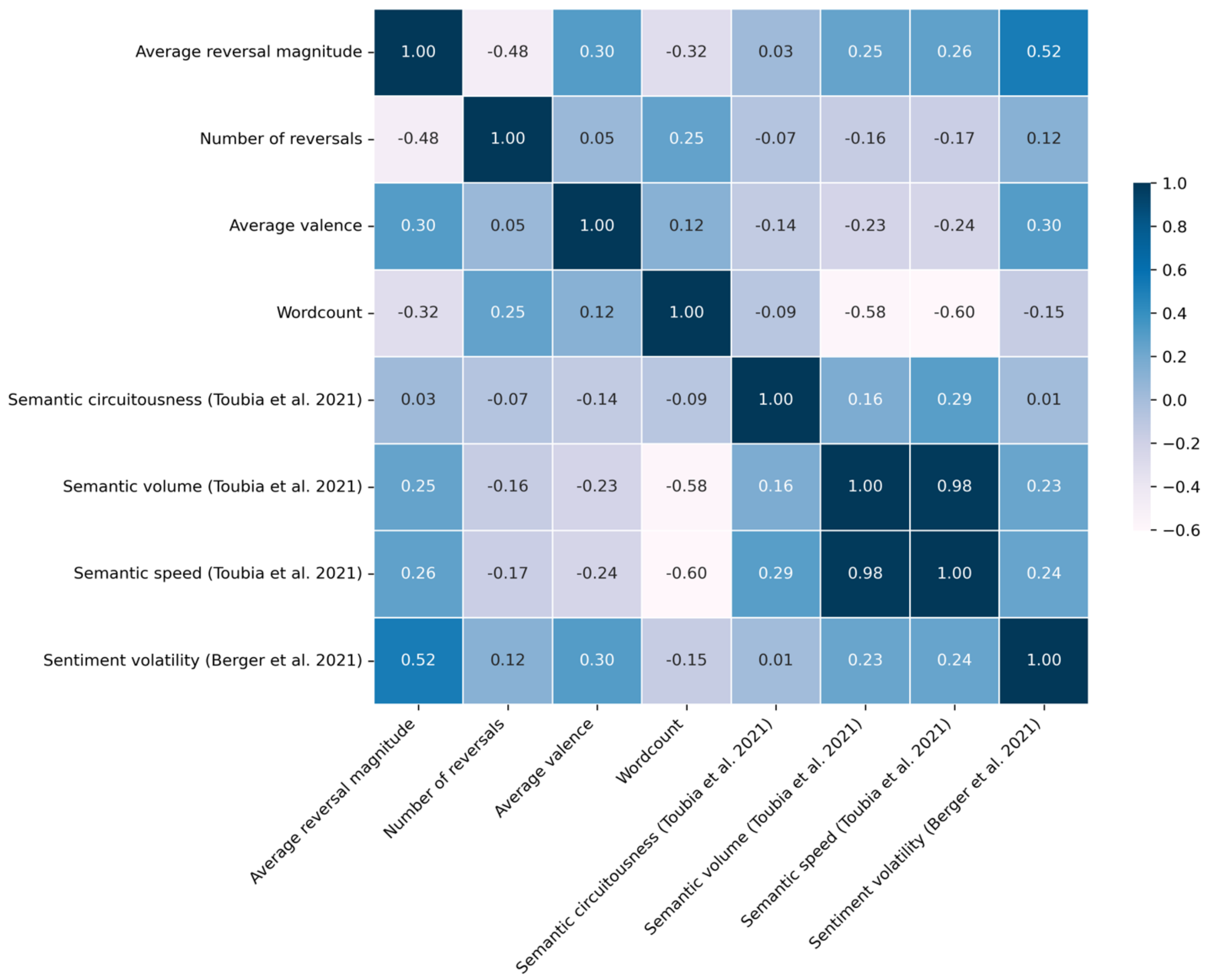

Appendix Figure S2—Correlation matrix between linear covariates of main specification for movies analysis. Based on sample of 3,713 movies. Circuitousness, volume and speed based on Toubia et al. (*13*), built using code shared from original study authors. Sentiment volatility based on Berger et al. (*30*), built based on method described in original paper.

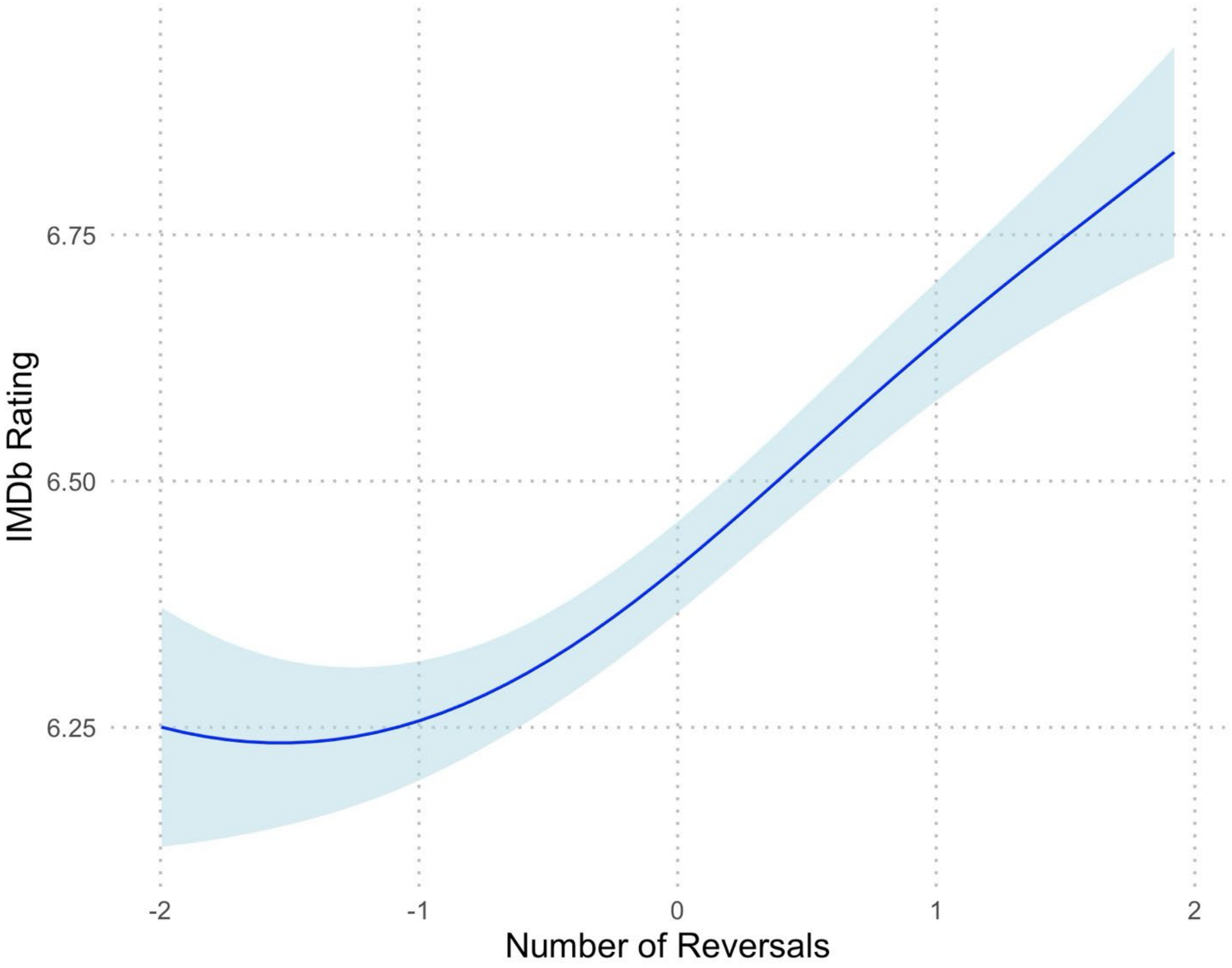

Appendix Figure S3—Generalized additive model of number of reversals effect curvature ±2 standard deviations from the mean. Based on specification (1) but allowing for smooth number of reversals term.

Study 2: TV Shows

Results

Alternative Measures of Valence

Results are similar when using alternative approaches to scoring valence: LabMT ($B_{num} = 0.057$, $t_{num}(18976) = 10.52,\ p_{num} < 0.001;\ B_{mag} = 0.066,\ t_{mag}(18976) = 12.17,\ p_{mag} < 0.001$) and the Evaluative Lexicon ($B_{num} = 0.066,\ t_{num}(12762) = 10.32, p_{num} < 0.001;\ B_{mag} = 0.077,\ t_{mag}(12762) = 11.98, p_{mag} < 0.001$).

Peak/End Effects

There were no significant effects of peak (*B* = 0.007, *t*(19334) = 1.12, *p* = 0.26) or end reversal magnitudes (*B* = 0.002, *t*(19334) = 0.26, *p* = 0.79). The number of reversals and the average magnitude of reversals remained significant (p_{num} < 0.001, p_{mag} = 0.001).

Effect Curvature

We also examined the limits to the number of reversals. Is it possible to have too many reversals in a narrative? To test this, we added a squared term to the model for the number of reversals. For TV, the results suggest that the effect of reversals diminishes as there is a larger number (*B* = -0.134, *t*(19336) = -4.55, *p* < 0.001), but this effect is not consistent across studies. We also inspected changing curvature of effects using a generalized additive model, as shown in Appendix Figure S12, and found that effects appeared to slope off at the upper end of the observed range, but again this effect is not found in the other studies.

Extreme Observations

In the histogram below, there is a small number of extreme observations. Results are similar when winsorizing these observations, defined as those three or more standard deviations from the mean ($B_{num} = 0.040$, $t_{num}(19336) = 6{,}97, p_{num} < 0.001;\ B_{mag} = 0.031, t_{mag}(19336) = 5.31, p_{mag} < 0.001$).

Cross-correlations

We present cross-correlations in Appendix Figure S5. There is again evidence of possible collinearity between semantic speed and volume from Toubia et al. (*13*). As in Study 1, all reversal measures remain significant for models that exclude either semantic speed or semantic volume (see Appendix Table S9).

Appendix Table S6

Reversals and TV Evaluations, Unrestricted Sample

Predictors	Reversals (1)	Controls (2)	Controls (3)
Number of reversals	0.043***	0.037***	0.020***
	(0.006)	(0.008)	(0.006)
Average reversal magnitude	0.033***	0.041***	0.026***
	(0.006)	(0.008)	(0.007)
**Controls**			
Average valence		-0.069***	-0.070***
		(0.007)	(0.006)
Subtitle wordcount		-0.024**	-0.017*
		(0.008)	(0.008)
Semantic circuitousness		-0.040***	-0.006
		(0.008)	(0.007)
Semantic volume		-0.125***	-0.147***
		(0.032)	(0.034)
Semantic speed		0.183***	0.147***
		(0.036)	(0.038)
Sentiment volatility		0.002	0.026***
		(0.008)	(0.007)
TV Show Fixed Effects	NO	NO	YES
Year Fixed Effects	NO	YES	YES
Genre Fixed Effects	NO	YES	YES
Maturity Rating Fixed Effects	NO	YES	YES
Constant	7.764***	8.642***	7.864***
	(0.005)	(0.678)	(0.416)
Adjusted R-squared	0.003	0.130	0.547
N. of TV episodes	19501	19501	18830

NOTES — *** p < 0.001, ** p < 0.01, * p < 0.05

Appendix Table S7

Reversals and TV Evaluations, Alternative Window Sizes

Window size	Smaller (2 min)	Larger (5 min)
Number of reversals	0.030***	0.020*
	(0.008)	(0.008)
Average reversal magnitude	0.014^{+}	0.032***
	(0.008)	(0.009)
**Controls**		
Average valence	-0.068***	-0.066***
	(0.007)	(0.007)
Subtitle wordcount	-0.020**	-0.019*
	(0.008)	(0.008)
Semantic circuitousness	-0.087***	-0.081***
	(0.010)	(0.010)
Semantic volume	-0.367***	-0.331***
	(0.050)	(0.050)
Semantic speed	0.451***	0.408***
	(0.054)	(0.054)
Sentiment volatility	0.018*	0.012
	(0.008)	(0.009)
Year Fixed Effects	YES	YES
Genre Fixed Effects	YES	YES
Maturity Rating Fixed Effects	YES	YES
Constant	8.664***	8.683***
	(0.674)	(0.676)
Adjusted R-squared	0.132	0.131
N. of TV episodes	19235	19371

NOTES — *** $p < 0.001$, ** $p < 0.01$, * $p < 0.05$, $^{+}$ $p < 0.10$

Appendix Table S8

Reversals and TV Evaluations, Fixed Window Overlap

Number of reversals	0.033***
	(0.007)
Average reversal magnitude	0.031***
	(0.008)
**Controls**	
Average valence	-0.079***
	(0.007)
Subtitle wordcount	-0.017*
	(0.008)
Semantic circuitousness	-0.086***
	(0.010)
Semantic volume	-0.368***
	(0.050)
Semantic speed	0.445***
	(0.055)
Sentiment volatility	0.009
	(0.007)
Year Fixed Effects	YES
Genre Fixed Effects	YES
Maturity Rating Fixed Effects	YES
Constant	8.672***
	(0.674)
Adjusted R-squared	0.133
N. of TV episodes	19207

NOTES — *** p < 0.001, ** p < 0.01, * p < 0.05

Window overlap fixed at the median overlap of the baseline specification.

Appendix Table S9

Reversals and TV Evaluations, Dropping Collinear Controls

	No "Speed" Control	No "Volume" Control
Number of reversals	0.035***	0.036***
	(0.008)	(0.008)
Average reversal magnitude	0.040***	0.040***
	(0.009)	(0.009)
**Controls**		
Average valence	-0.068***	-0.067***
	(0.007)	(0.007)
Subtitle wordcount	-0.028***	-0.021**
	(0.008)	(0.008)
Semantic circuitousness	-0.013*	-0.023***
	(0.005)	(0.006)
Semantic volume	0.040***	.
	(0.009)	(.)
Semantic speed	.	0.055***
	(.)	(0.009)
Sentiment volatility	0.004	0.003
	(0.008)	(0.008)
Year Fixed Effects	YES	YES
Genre Fixed Effects	YES	YES
Maturity Rating Fixed Effects	YES	YES
Constant	8.610***	8.622***
	(0.676)	(0.676)
Adjusted R-squared	0.129	0.130
N. of TV episodes	19339	19339

NOTES — *** p < 0.001, ** p < 0.01, * p < 0.05

Appendix Table S10

Summary Statistics for TV Sample

	Mean	Std. Dev.	Median
Average reversal magnitude	0.0610	0.0204	0.0583
Number of reversals	11.9066	2.9084	12.0000
Average valence	0.1377	0.0314	0.1345
Wordcount	4.3727	2.0187	4.1230
Semantic circuitousness	0.1284	0.0195	0.1262
Semantic volume	0.3707	0.0657	0.3620
Semantic speed	0.6809	0.1247	0.6616
Sentiment volatility	0.0238	0.0082	0.0226

NOTES — Unstandardized summary statistics for linear covariates in TV sample used in Study 2, based on 19,339 TV shows. Wordcount measured in units of 1000 words.

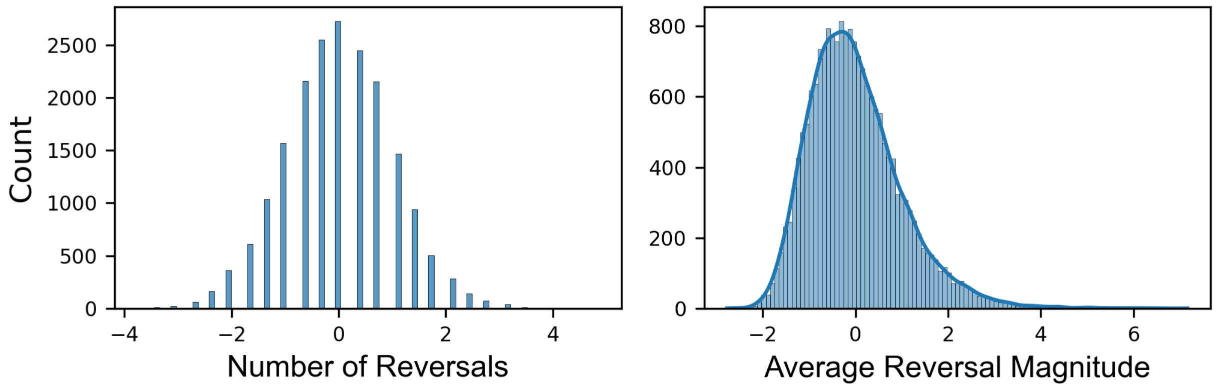

Appendix Figure S4—Histograms of standardized number of reversals and standardized average reversal magnitude for television show sample used in study 2.

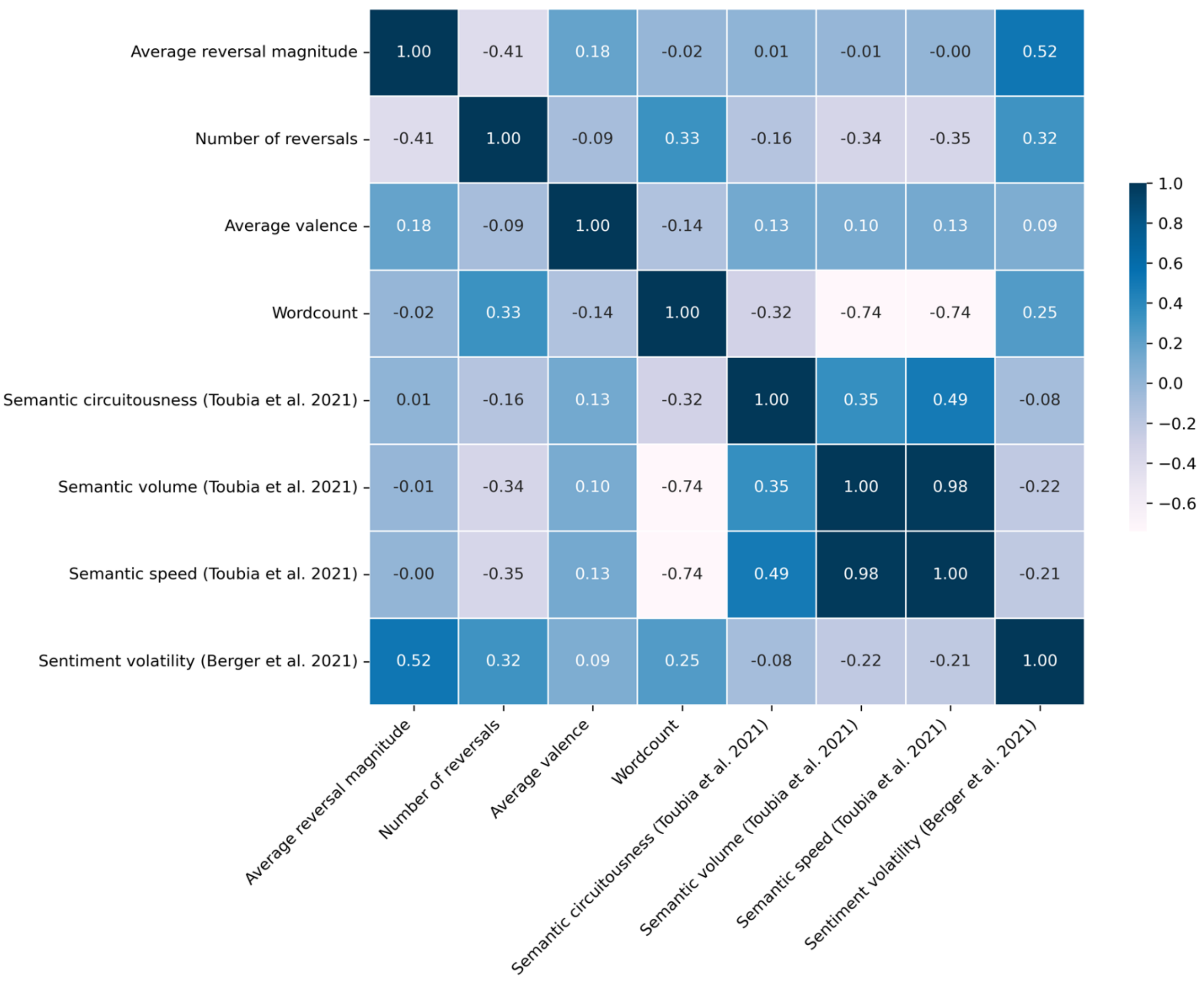

Appendix Figure S5—Correlation matrix between linear covariates of main specification for TV show analysis. Based on sample of 19,339 TV shows. Circuitousness, volume and speed based on Toubia et al. (*13*), built using code shared from original study authors. Sentiment volatility based on Berger et al. (*30*), built based on method described in original paper.

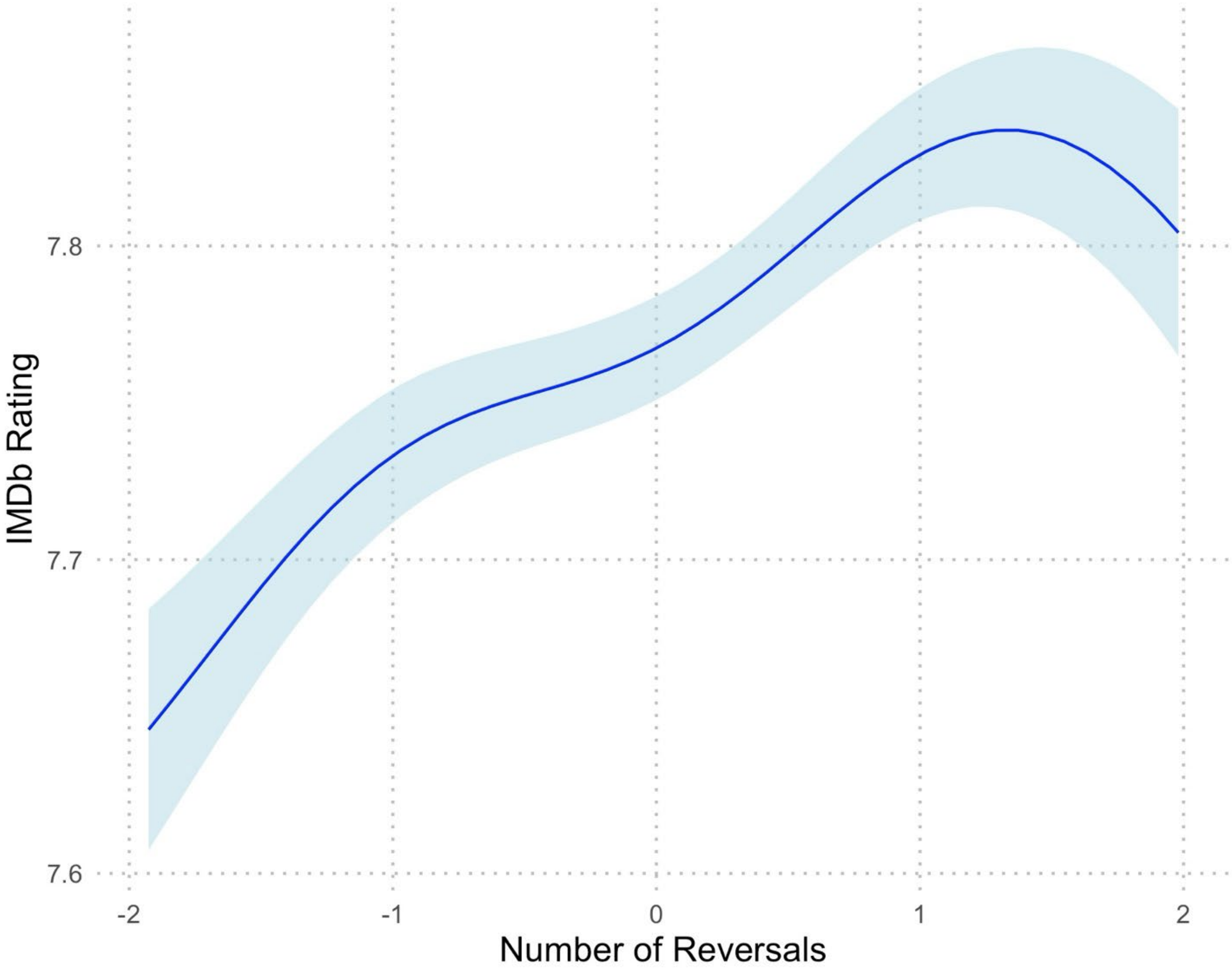

Appendix Figure S6—Generalized additive model of number of reversals effect curvature ±2 standard deviations from the mean. Based on specification (1) but allowing for smooth number of reversals term.

Study 3: Novels

Methods

Genre Controls

We determined genres for Gutenberg books with the support of a research assistant who coded the following genres for novels to approximately match the genres we observe in our TV and movie samples: War, Biography, Romance, Drama, Fantasy, Family, Science Fiction, Action, Thriller, Western, Horror, Mystery, Crime, History, Periodicals, and Others.

Results

Alternative Measures of Valence

Results are similar when using alternative approaches to scoring valence: LabMT ($B_{num} = 0.225,\ t_{num}(8660) = 15.71, p_{num} < 0.001;\ B_{mag} = 0.183, t_{mag}(8660) = 12.78,\ p_{mag} < 0.001$) and the Evaluative Lexicon ($B_{num} = 0.236,\ t_{num}(8240) = 15.74, p_{num} < 0.001;\ B_{mag} = 0.174, t_{mag}(8240) = 11.63,$ $p_{mag} < 0.001$).

Peak/End Effects

There were no significant effects of peak ($B = 0.043$, $t(8660) = 0.05$, $p = 0.959$) or end reversal magnitudes ($B = -0.894$, $t(8660) = -0.94$, $p = 0.350$). The number of reversals and the average magnitude of reversals remained significant ($p_{num} < 0.001$, $p_{mag} < 0.001$).

Effect Curvature

We added a squared term to the model for the number of reversals. For novels, the results suggest that the effect of reversals does not have any significant curvature in either direction ($B = 0.001$, $t(8660) = 0.89$, $p = 0.373$). A generalized additive model, as shown in Appendix Figure S9, also showed a linear effect.

Extreme Observations

In the histogram below, there is a small number of extreme observations. Results are similar when winsorizing these observations, defined as those three or more standard deviations from the mean ($B_{num} = 0.194,$ $t_{num}(8660) = 12.69, p_{num} < 0.001;\ B_{mag} = 0.202, t_{mag}(8660) = 12.61, p_{mag} < 0.001$).

Cross-correlations

We present cross-correlations in Appendix Figure S8. There is again evidence of possible collinearity between semantic speed and volume from Toubia et al. (*13*). As in previous studies, all reversal measures remain significant for models that exclude either semantic speed or semantic volume (see Appendix Table S14).

Appendix Table S11

Reversals and Novel Downloads, 25,000 Minimum Total Wordcount

Predictors	Reversals (1)	Controls (2)	Controls (3)
Number of reversals	0.150***	0.068***	0.062***
	(0.012)	(0.016)	(0.016)
Average reversal magnitude	0.169***	0.097***	0.089***
	(0.012)	(0.018)	(0.018)
**Controls**			
Average valence		-0.158***	-0.162***
		(0.012)	(0.012)
Novel wordcount		0.098***	0.092***
		(0.016)	(0.016)
Semantic circuitousness		0.035	0.024
		(0.024)	(0.023)
Semantic volume		0.454***	0.371**
		(0.118)	(0.117)
Semantic speed		-0.452***	-0.383***
		(0.107)	(0.106)
Sentiment volatility		0.122***	0.109***
		(0.021)	(0.022)
Author Decade-of-Birth Fixed Effects	NO	NO	YES
Genre Fixed Effects	NO	YES	YES
Constant	3.015***	3.082***	3.060***
	(0.011)	(0.048)	(0.048)
Adjusted R-squared	0.019	0.131	0.160
N. of novels	11554	11554	11554

NOTES — *** $p < 0.001$, ** $p < 0.01$, * $p < 0.05$

Appendix Table S12

Reversals and Novel Downloads, Alternative Window Sizes

Window size	Smaller (8,000 words)	Larger (12,000 words)
Number of reversals	0.055**	0.059**
	(0.020)	(0.019)
Average reversal magnitude	0.054**	0.093***
	(0.020)	(0.020)
**Controls**		
Average valence	-0.186***	-0.176***
	(0.014)	(0.014)
Novel wordcount	0.062**	0.090***
	(0.019)	(0.018)
Semantic circuitousness	0.027	0.018
	(0.028)	(0.028)
Semantic volume	0.344**	0.262*
	(0.108)	(0.107)
Semantic speed	-0.369***	-0.311***
	(0.095)	(0.094)
Sentiment volatility	0.173***	0.106***
	(0.025)	(0.022)
Genre Fixed Effects	YES	YES
Constant	3.208***	3.208***
	(0.055)	(0.055)
Adjusted R-squared	0.131	0.127
N. of novels	8663	8663

NOTES — *** $p < 0.001$, ** $p < 0.01$, * $p < 0.05$

Appendix Table S13

Reversals and Novel Downloads, Fixed Window Overlap

Number of reversals	0.060**
	(0.019)
Average reversal magnitude	0.059**
	(0.020)
**Controls**	
Average valence	-0.175***
	(0.014)
Novel wordcount	0.124***
	(0.017)
Semantic circuitousness	-0.010
	(0.029)
Semantic volume	0.108
	(0.106)
Semantic speed	-0.213*
	(0.095)
Sentiment volatility	0.046**
	(0.016)
Genre Fixed Effects	YES
Constant	3.203***
	(0.055)
Adjusted R-squared	0.124
N. of novels	8495

NOTES — *** p < 0.001, ** p < 0.01, * p < 0.05

Window overlap fixed at the median overlap of the baseline specification.

Appendix Table S14

Reversals and Novel Downloads, Dropping Collinear Controls

	No "Speed" Control	No "Volume" Control
Number of reversals	0.071***	0.070***
	(0.019)	(0.019)
Average reversal magnitude	0.077***	0.077***
	(0.020)	(0.020)
**Controls**		
Average valence	-0.180***	-0.181***
	(0.014)	(0.014)
Subtitle wordcount	0.073***	0.069***
	(0.019)	(0.019)
Semantic circuitousness	-0.066***	-0.050***
	(0.014)	(0.013)
Semantic volume	-0.069***	.
	(0.019)	(.)
Semantic speed	.	-0.071***
	(.)	(0.017)
Sentiment volatility	0.130***	0.131***
	(0.024)	(0.023)
Genre Fixed Effects	YES	YES
Constant	3.204***	3.204***
	(0.055)	(0.055)
Adjusted R-squared	0.129	0.130
N. of novels	8663	8663

NOTES — *** p < 0.001, ** p < 0.01, * p < 0.05

Appendix Table S15

Summary Statistics for Novels Sample

	Mean	Std. Dev.	Median
Average reversal magnitude	0.0175	0.0081	0.0161
Number of reversals	9.9192	3.1373	10.0000
Average valence	0.1360	0.0225	0.1351
Wordcount	96.7123	70.3890	82.1330
Semantic circuitousness	0.1267	0.0289	0.1247
Semantic volume	0.1012	0.0157	0.1021
Semantic speed	0.1952	0.0259	0.1965
Sentiment volatility	0.0054	0.0019	0.0051

NOTES — Unstandardized summary statistics for linear covariates in novels sample used in Study 3, based on 8,663 novels. Wordcount measured in units of 1000 words.

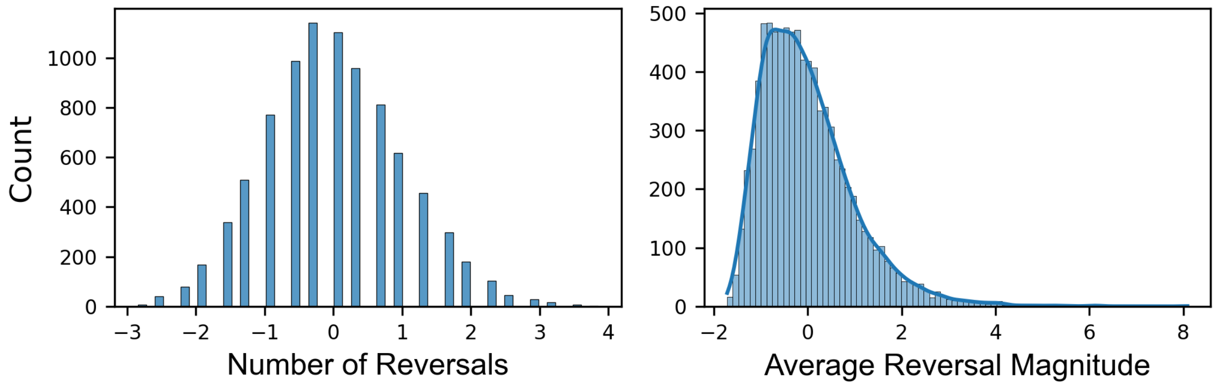

Appendix Figure S7—Histograms for standardized number of reversals and standardized average reversal magnitude for novel sample.

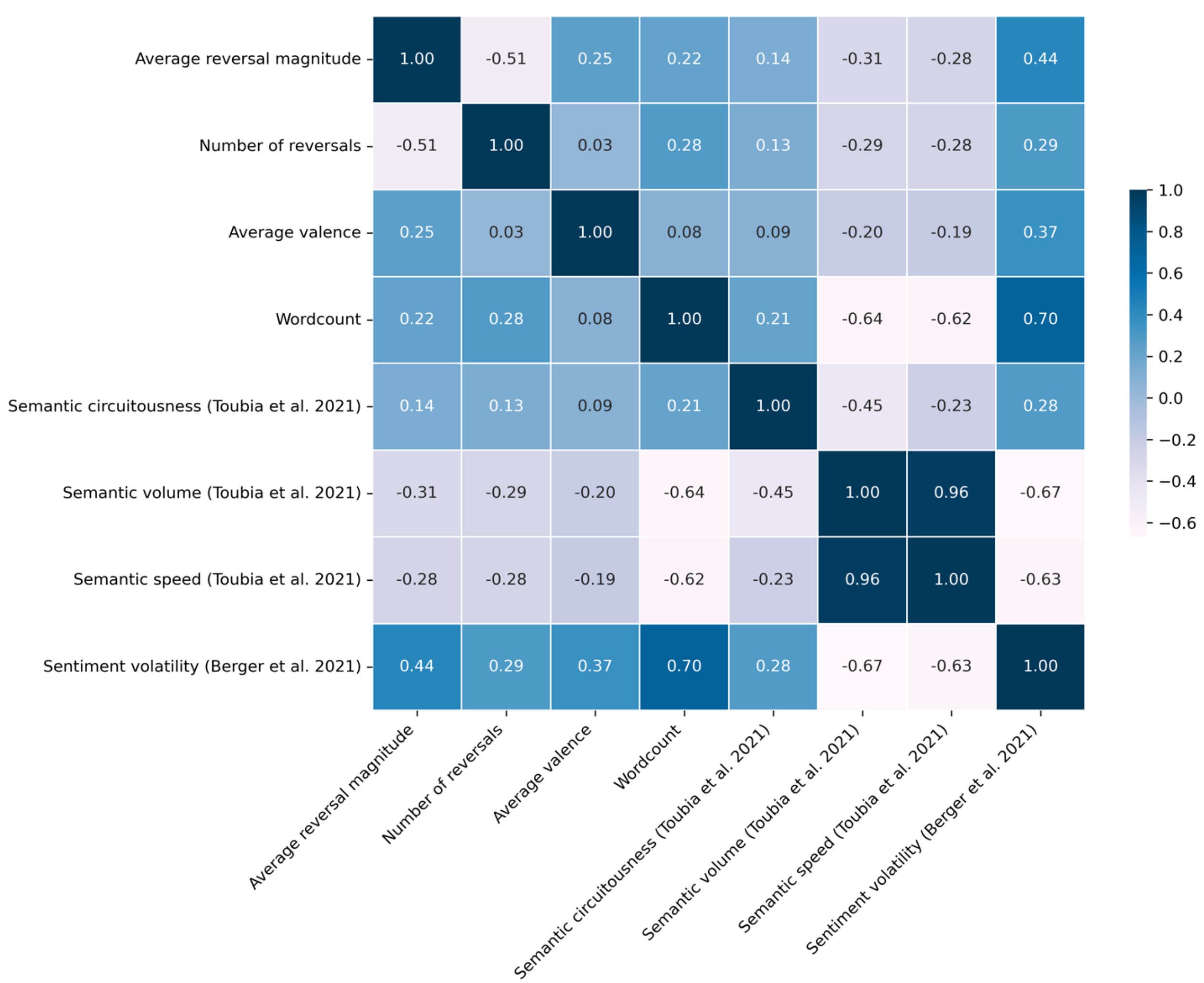

Appendix Figure S8—Correlation matrix between linear covariates of main specification for TV show analysis. Based on sample of 8,663 novels. Circuitousness, volume and speed based on Toubia et al. (*13*), built using code shared from original study authors. Sentiment volatility based on Berger et al. (*30*), built based on method described in original paper.

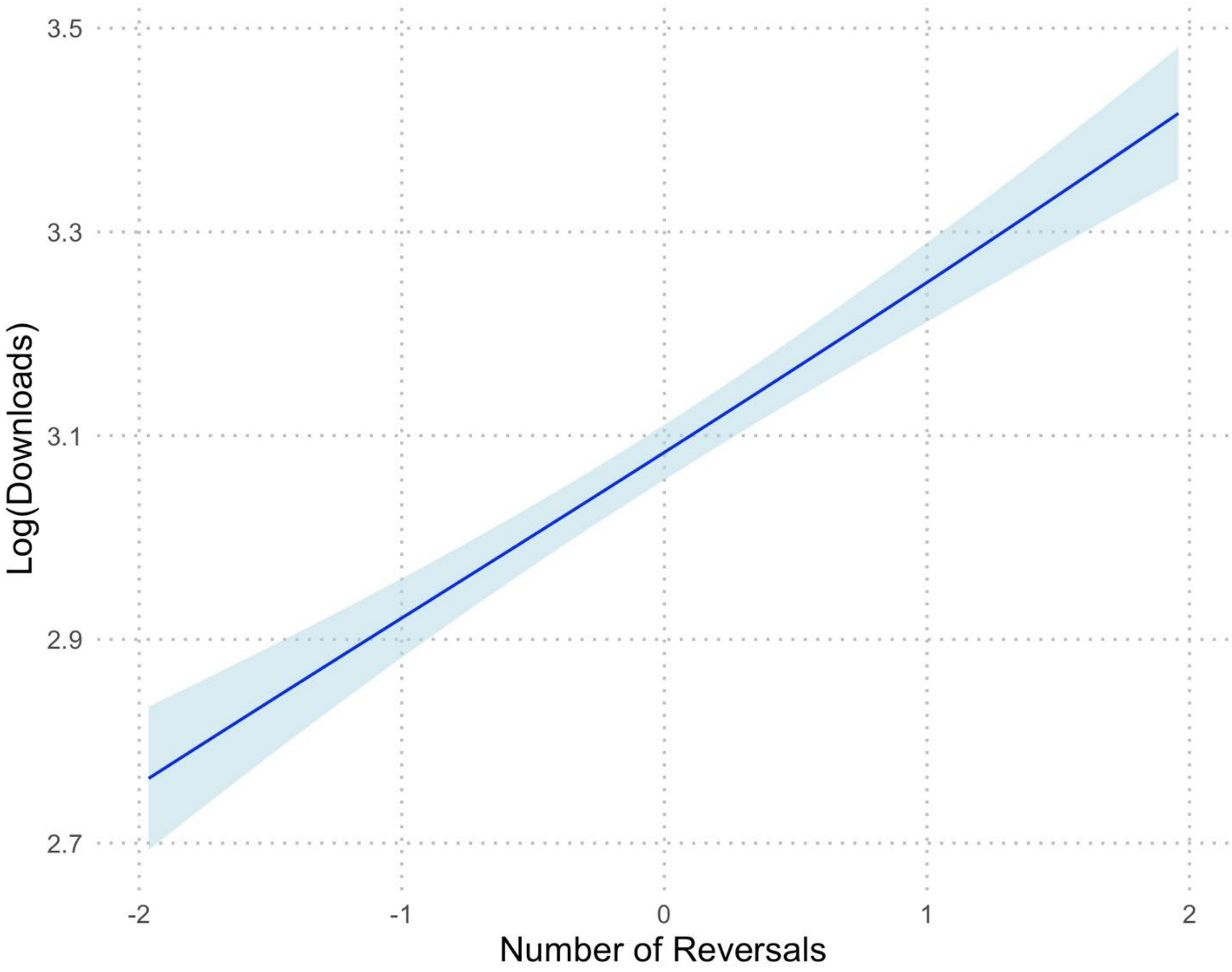

Appendix Figure S9—Generalized additive model of number of reversals effect curvature ±2 standard deviations from the mean. Based on specification (1) but allowing for smooth number of reversals term.

Study 4: Fundraising Pitches

Methods

Details of GoFundMe Translation

While the large majority of fundraisers (83%) in our GoFundMe sample are English-language, a small proportion are not in English. For these, we use Google Translate to translate these fundraising pitches into English before scoring the pitches' time series of valence, through the Google Translate API (*40*). Results are also similar when using only fundraisers that are originally written in English (see Appendix Table S17).

Results

Alternative Measures of Valence

Given the much shorter length of the GoFundMe texts as well as the considerably smaller sample size compared to the previous contexts, the GoFundMe results are more sensitive toward the measurement approach used. Nevertheless, the results are directionally consistent with the primary results, though ultimately non-significant. We report the results here for the sake of full transparency: LabMT ($B_{num} = 0.038,\ z_{num} = 0.35, p_{num} = 0.73;\ B_{mag} = 0.104,\ z_{mag} = 1.13, p_{mag} = 0.26$) and the Evaluative Lexicon ($B_{num} = 0.082,\ z_{num} = 0.75, p_{num} = 0.45;\ B_{mag} = 0.060, z_{mag} = 0.55,\ p_{mag} = 0.58$).

Peak/End Effects

There were no significant effects of peak reversal magnitudes (B = -0.195, z = -1.627, p = 0.104), but there was a significant effect of end reversal magnitudes (B = 0.261, z = 2.22, p = 0.026). The estimated effect of the number of reversals and average magnitude of reversals both remain significant (p_{num} = 0.009, p_{mag} = 0.027).

Effect Curvature

We added a squared term to the model for the number of reversals. For fundraisers, the results suggest that the effect of reversals potentially decreases as there is a larger number, but this effect is not significant and not consistent across all studies (B = -0.276, z = -0.734, p = 0.463). A generalized additive model, as shown in Appendix Figure S12, also showed a linear effect.

Extreme Observations

In the histogram below, there is a small number of extreme observations. Results are similar when winsorizing these observations, defined as those three or more standard deviations from the mean ($B_{num} = 0.281, z = 2.33, p_{num} = 0.020;\ B_{mag} = 0.349, z = 2.86, p_{mag} = 0.004$).

Cross-correlations

We present cross-correlations in Appendix Figure S11. There is again evidence of possible collinearity between semantic speed and volume from Toubia et al. (*13*). As in previous studies, all reversal measures remain significant for models that exclude either semantic speed or semantic volume (see Appendix Table S20).

Appendix Table S16

Reversals and Fundraiser Success, Restricted to Fundraisers That Had Been Online for 30 Days or More

Predictors	Reversals (1)	Controls (2)
Number of reversals	0.228^{+}	0.320*
	(0.118)	(0.154)
Average reversal magnitude	0.307**	0.356*
	(0.108)	(0.156)
**Controls**		
Average valence		0.136
		(0.143)
Fundraiser wordcount		0.025
		(0.216)
Number of months online		0.205*
		(0.084)
English-language		-0.333
		(0.339)
Semantic circuitousness		-0.131
		(0.285)
Semantic volume		0.129
		(0.612)
Semantic speed		0.061
		(0.606)
Sentiment volatility		-0.058
		(0.192)
Category Fixed Effects	NO	YES
Constant	-2.065***	-0.675
	(0.098)	(1.204)
N. of fundraisers	1061	1061
Pseudo R-squared	0.010	0.068

NOTES — *** $p < 0.001$, ** $p < 0.01$, * $p < 0.05$, $^{+}$ $p < 0.10$

Appendix Table S17

Reversals and Fundraiser Success, Restricted to Only English-Language

Predictors	Reversals (1)	Controls (2)
Number of reversals	0.288*	0.368*
	(0.125)	(0.166)
Average reversal magnitude	0.275*	0.301^{+}
	(0.118)	(0.178)
**Controls**		
Average valence		0.149
		(0.156)
Fundraiser wordcount		-0.208
		(0.281)
Number of months online		0.249**
		(0.089)
English-language		0.000
		(.)
Semantic circuitousness		-0.240
		(0.374)
Semantic volume		-0.146
		(0.884)
Semantic speed		0.152
		(0.982)
Sentiment volatility		-0.027
		(0.214)
Category Fixed Effects	NO	YES
Constant	-2.096***	-0.761
	(0.105)	(1.239)
N. of fundraisers	947	947
Pseudo R-squared	0.010	0.074

NOTES — *** $p < 0.001$, ** $p < 0.01$, * $p < 0.05$, $^{+}$ $p < 0.10$

Appendix Table S18

Reversals and Fundraiser Success, Alternative Window Sizes

Window Size	Smaller (450 words)	Larger (550 words)
Number of reversals	0.136	0.263^{+}
	(0.147)	(0.150)
Average reversal magnitude	0.227	0.263
	(0.141)	(0.161)
**Controls**		
Average valence	0.166	0.220
	(0.149)	(0.138)
Fundraiser wordcount	0.042	0.054
	(0.215)	(0.209)
Number of months online	0.235**	0.231**
	(0.080)	(0.081)
English-language	-0.180	-0.147
	(0.336)	(0.341)
Semantic circuitousness	-0.080	-0.074
	(0.266)	(0.253)
Semantic volume	0.236	0.184
	(0.587)	(0.570)
Semantic speed	-0.135	-0.147
	(0.583)	(0.563)
Sentiment volatility	-0.069	-0.197
	(0.203)	(0.196)
Category Fixed Effects	YES	YES
Constant	-0.834	-0.790
	(1.200)	(1.200)
N. of fundraisers	1133	1132
Pseudo R-squared	0.062	0.063

NOTES — *** $p < 0.001$, ** $p < 0.01$, * $p < 0.05$, $^{+}$ $p < 0.10$

Appendix Table S19

Reversals and Fundraiser Success, Fixed Window Overlap

Number of reversals	0.161
	(0.150)
Average reversal magnitude	0.231^{+}
	(0.132)
**Controls**	
Average valence	0.120
	(0.124)
Fundraiser wordcount	-0.004
	(0.195)
Number of months online	0.236**
	(0.080)
English-language	-0.309
	(0.348)
Semantic circuitousness	-0.102
	(0.271)
Semantic volume	0.162
	(0.593)
Semantic speed	-0.138
	(0.587)
Sentiment volatility	-0.053
	(0.115)
Category Fixed Effects	YES
Constant	-0.805
	(1.206)
N. of fundraisers	1134
Pseudo R-squared	0.061

NOTES — *** $p < 0.001$, ** $p < 0.01$, * $p < 0.05$, $^{+}$ $p < 0.10$

Window overlap fixed at the median overlap of the baseline specification.

Appendix Table S20

Reversals and Fundraiser Success, Dropping Collinear Controls

	No “Speed” Control	No “Volume” Control
Number of reversals	0.368*	0.368*
	(0.151)	(0.151)
Average reversal magnitude	0.376*	0.375*
	(0.153)	(0.153)
**Controls**		
Average valence	0.127	0.128
	(0.144)	(0.144)
Fundraiser wordcount	-0.009	-0.022
	(0.214)	(0.216)
Number of months online	0.226**	0.226**
	(0.081)	(0.081)
English-language	-0.274	-0.260
	(0.334)	(0.333)
Semantic circuitousness	-0.128	-0.183
	(0.147)	(0.139)
Semantic volume	0.134	.
	(0.212)	(.)
Semantic speed	.	0.114
	(.)	(0.211)
Sentiment volatility	-0.077	-0.078
	(0.191)	(0.192)
Category Fixed Effects	YES	YES
Constant	-0.738	-0.727
	(1.203)	(1.205)
N. of fundraisers	1133	1133
Pseudo R-squared	0.067	0.067

NOTES — *** p < 0.001, ** p < 0.01, * p < 0.05

Appendix Table S21

Summary Statistics for Fundraiser Sample

	Mean	Std. Dev.	Median
Average reversal magnitude	0.0291	0.0199	0.0230
Number of reversals	7.3813	3.1012	7.0000
Average valence	0.1128	0.0429	0.1069
Wordcount	1.4863	0.6966	1.2570
Number of months online	9.9287	17.8172	2.7667
English language	0.8358	0.3706	1.0000
Semantic circuitousness	0.1734	0.0615	0.1607
Semantic volume	0.5325	0.0802	0.5483
Semantic speed	1.0151	0.1418	1.0454
Sentiment volatility	0.0066	0.0028	0.0059

NOTES — Unstandardized summary statistics for linear covariates in fundraiser sample used in Study 4, based on 1,133 GoFundMe fundraisers. Wordcount measured in units of 1000 words.

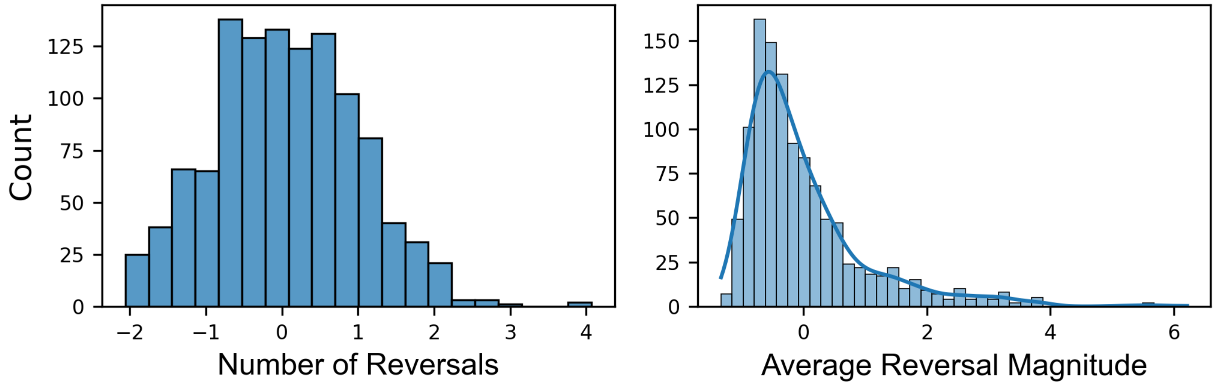

Appendix Figure S10—Histogram of standardized number of reversals and standardized average reversal magnitude for GoFundMe sample.

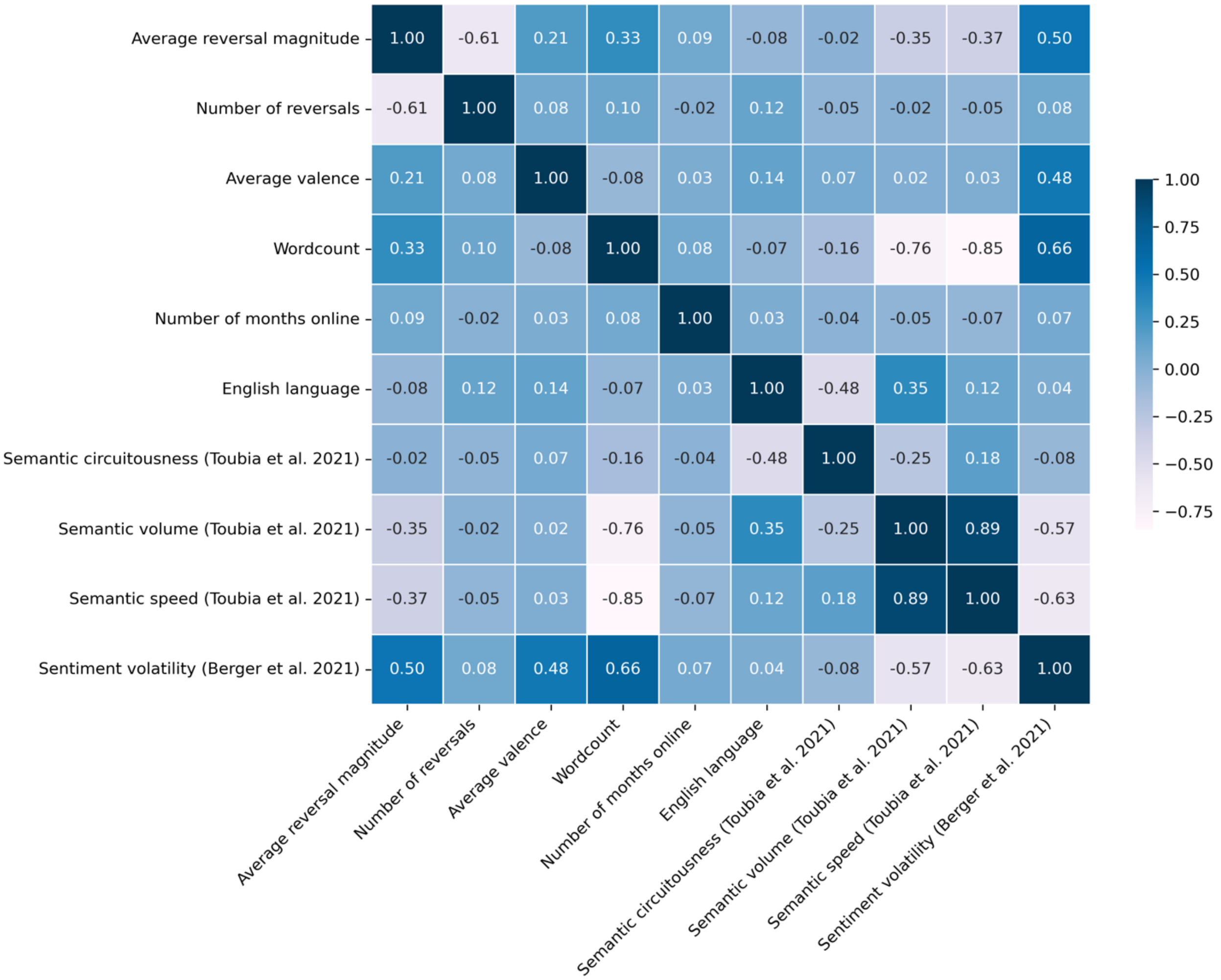

Appendix Figure S11—Correlation matrix between linear covariates of main specification for TV show analysis. Based on sample of 1,133 fundraisers. Circuitousness, volume and speed based on Toubia et al. (*13*), built using code shared from original study authors. Sentiment volatility based on Berger et al. (*30*), built based on method described in original paper.

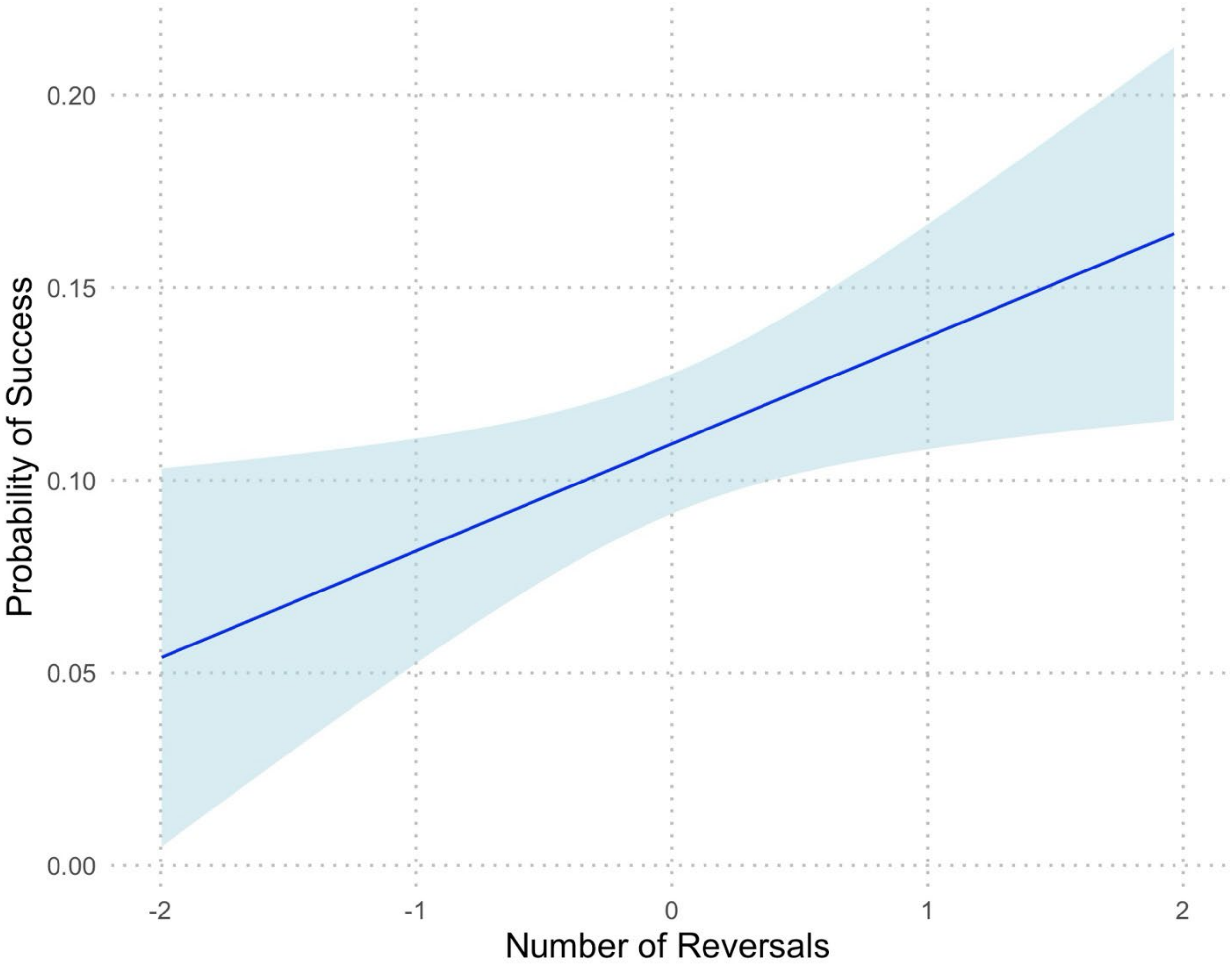

Appendix Figure S12—Generalized additive model of number of reversals effect curvature ±2 standard deviations from the mean. Based on specification (1) but allowing for smooth number of reversals term.

Ancillary Experiment: Narrative Reversal Validation

We have based our mapping of valence reversals to narrative reversals, or turning points, as drawn from dramaturgical theory. In this section, we present evidence to support this mapping on the basis of readers' perceptions of high-reversal versus low-reversal GoFundMe fundraising pitches. We hypothesized that readers would perceive pitches with both more reversals and larger reversals as having a greater presence of turning points in a narrative.

To test this hypothesis, we showed participants high- or low-reversal GoFundMe passages. Internal analyses indicated that passages with differing magnitudes of reversals also showed natural covariation with the overall valence of the passage and with length. To ensure passages differed only in the magnitude of their reversals, we controlled for average valence and wordcount. We then selected passages in the top and bottom third in terms of the number and average size of reversals. Three passages of each were randomly selected (six total) and these represented high reversal and low reversal passages, respectively.

Participants (N = 200; M_{age} = 41.46, 51% female) were randomly assigned to read either a high- or low-reversal passage and then were asked four questions to assess their perceptions of the magnitude of reversals in that passage. Specifically, they were asked about the extent to which there were 1) turning points presented in the passage, 2) key events or occurrences that changed the direction of the passage, 3) crossroads that altered the direction of the passage, and 4) critical points or critical moments that changed the outcome of the passage (1: Not at all, 7: A great deal). We averaged these four items together to create a single index of the magnitude of narrative reversals (α = .91; *M* = 4.79, *SD* = 1.33). Higher numbers indicate a greater presence of turning points in the narrative.

An independent t-test comparing the high- vs. low-reversal passages revealed a significant difference between conditions in the perceived magnitude of narrative reversals. Specifically, high-reversal passages (*M* = 5.09, *SD* = 1.15) were perceived as having significantly greater reversal characteristics compared to low-reversal passages (*M* = 4.49, *SD* = 1.43; $t(192.33) = 3.29$, $p = .001$). These results support the mapping of valence reversals to narrative turning points, consistent with dramaturgical theory.

REFERENCES AND NOTES

1. J. S. Bruner, *Acts of Meaning: Four Lectures on Mind and Culture* (Harvard Univ. Press, 1990).

2. R. I. M. Dunbar, *Grooming, Gossip, and the Evolution of Language* (Harvard Univ. Press, 1996).

3. R. F. Baumeister, L. Zhang, K. D. Vohs, Gossip as cultural learning. *Rev. Gen. Psychol.* **8**, 111–121 (2004).

4. R. A. Mar, K. Oatley, The function of fiction is the abstraction and simulation of social experience. *Perspect. Psychol. Sci.* **3**, 173–192 (2008).

5. A. Piper, Computational narrative understanding: A big picture analysis, in *Proceedings of the Big Picture Workshop*, Y. Elazar, A. Ettinger, N. Kassner, S. Ruder, N. A. Smith, Eds. (Association for Computational Linguistics, Singapore, 2023), pp. 28–39; https://aclanthology.org/2023.bigpicture-1.3.

6. G. Freytag, *Technique of the Drama: An Exposition of Dramatic Composition and Art*, S. Griggs, Ed. (1895).

7. J. Gao, M. L. Jockers, J. Laudun, T. Tangherlini, A multiscale theory for the dynamical evolution of sentiment in novels, in *2016 International Conference on Behavioral, Economic and Socio-Cultural Computing* (*BESC*) (2016), pp. 1–4.

8. B. M. Schmidt, Plot arceology: A vector-space model of narrative structure, in *2015 IEEE International Conference on Big Data* (*Big Data*) (2015), pp. 1667–1672.

9. A. J. Reagan, L. Mitchell, D. Kiley, C. M. Danforth, P. S. Dodds, The emotional arcs of stories are dominated by six basic shapes. *EPJ Data Sci.* **5**, 31 (2016).

10. R. L. Boyd, K. G. Blackburn, J. W. Pennebaker, The narrative arc: Revealing core narrative structures through text analysis. *Sci. Adv.* **6**, eabS2196 (2020).

11. G. Prince, *Narratology: The Form and Functioning of Narrative* (Walter de Gruyter, 2012).

12. J. Ouyang, K. McKeown, Modeling reportable events as turning points in narrative, in *Proceedings of the 2015 Conference on Empirical Methods in Natural Language Processing*, L. Màrquez, C. Callison-Burch, J. Su, Eds. (Association for Computational Linguistics, Lisbon, Portugal, 2015), pp. 2149–2158; https://aclanthology.org/D15-1257.

13. O. Toubia, J. Berger, J. Eliashberg, How quantifying the shape of stories predicts their success. *Proc. Natl. Acad. Sci. U.S.A.* **118**, e2011695118 (2021).

14. H. Laurino Dos Santos, J. Berger, The speed of stories: Semantic progression and narrative success. *J. Exp. Psychol. Gen.* **151**, 1833–1842 (2022).

15. J. Bruner, The narrative construction of reality. *Crit. Inq.* **18**, 1–21 (1991).

16. D. Herman, *Basic Elements of Narrative* (John Wiley & Sons, 2009).

17. P. Hühn, Event and eventfulness, in *Handbook of Narratology*, P. Hühn, J. C. Meister, J. Pier, W. Schmid, Eds. (De Gruyter, Boston, 2014), pp. 159–178.

18. J. Gottschall, *The Storytelling Animal: How Stories Make Us Human* (Houghton Mifflin Harcourt, 2012).

19. Aristotle, *Poetics* (Penguin Classics, New York, N.Y., New Ed edition., 1997).

20. E. M. Forster, *Aspects of the Novel* (Harcourt, Brace, 1927).

21. L. Katz, *Cleaning Augean Stables: Examining Drama's Strategies* (CreateSpace Independent Publishing Platform, Print Edition., 2012).

22. R. McKee, *Story: Substance, Structure, Style and the Principles of Screenwriting* (ReganBooks, New York, 1st edition., 1997).

23. K. Vonnegut, Shapes of Stories (1995); https://youtube.com/watch?v=oP3c1h8v2ZQ.

24. W. F. Brewer, E. H. Lichtenstein, Stories are to entertain: A structural-affect theory of stories. *J. Pragmat.* **6**, 473–486 (1982).

25. J. Ely, A. Frankel, E. Kamenica, Suspense and surprise. *J. Polit. Econ.* **123**, 215–260 (2015).

26. C. J. Hutto, E. Gilbert, VADER: A parsimonious rule-based model for sentiment analysis of social media text, in *Eighth International AAAI Conference on Weblogs and Social Media* (2014), vol. 8, pp. 216–225; https://ojs.aaai.org/index.php/ICWSM/article/view/14550.

27. P. S. Dodds, K. D. Harris, I. M. Kloumann, C. A. Bliss, C. M. Danforth, Temporal patterns of happiness and information in a global social network: Hedonometrics and twitter. *PLOS ONE* **6**, e26752 (2011).

28. M. D. Rocklage, R. H. Fazio, The evaluative lexicon: Adjective use as a means of assessing and distinguishing attitude valence, extremity, and emotionality. *J. Exp. Soc. Psychol.* **56**, 214–227 (2015).

29. M. D. Rocklage, D. D. Rucker, L. F. Nordgren, The evaluative lexicon 2.0: The measurement of emotionality, extremity, and valence in language. *Behav. Res.* **50**, 1327–1344 (2018).

30. J. Berger, Y. D. Kim, R. Meyer, What makes content engaging? How emotional dynamics shape success *J. Consum. Res.* **48**, 235–250 (2021).

31. A. Bartolome del Canto, Trendet (2020); https://github.com/alvarobartt/trendet.

32. D. Kahneman, B. L. Fredrickson, C. A. Schreiber, D. A. Redelmeier, When more pain is preferred to less: Adding a better end. *Psychol. Sci.* **4**, 401–405 (1993).

33. M. Gerlach, F. Font-Clos, A standardized project Gutenberg corpus for statistical analysis of natural language and quantitative linguistics. *Entropy* **22**, 126 (2020).

34. B. Atwood, How many words in a novel? (Guide to 18 Genres) (2022); https://thewritelife.com/how-many-words-in-a-novel/.

35. M. Beeson, How many pages in a novel, Novella, Novelette and Short Story?, *Weekend Publisher* (2022); https://weekendpublisher.com/how-many-pages-in-a-novel/.

36. Word Count Guide: How Long Is a Book, Short Story, or Novella? – 2023, *MasterClass* (2023). https://masterclass.com/articles/word-count-guide.

37. B. Armas, 12 tips to help you write a powerful GoFundMe fundraiser story, *GoFundMe* (2015); https://gofundme.com/c/blog/campaign-story.

38. Social Security Administration, Top Names Over the Last 100 Years (2022); https://ssa.gov/oact/babynames/decades/century.html.

39. M. D. Rocklage, D. D. Rucker, Text analysis in consumer research: An overview and tutorial, in *Handbook of Research Methods in Consumer Psychology*, F. R. Kardes, P. M. Herr, N. Schwarz, Eds. (Routledge, New York, NY, 2019), pp. 385–402.

40. Google Translate (2023); https://translate.google.com/.
